# What is the role of the film viewer? The effects of narrative comprehension and viewing task on gaze control in film

**DOI:** 10.1186/s41235-017-0080-5

**Published:** 2017-11-22

**Authors:** John P. Hutson, Tim J. Smith, Joseph P. Magliano, Lester C. Loschky

**Affiliations:** 1 Department of Psychological Sciences, Kansas State University, 492 Bluemont Hall, 1100 Mid-campus Dr, Manhattan, KS 66506 USA; 2 Department of Psychological Sciences, Birkbeck, University of London, Malet St, London, WC1E 7HX UK; 3 Department of Psychology, Northern Illinois University, 361 Psychology-Computer Science Building, DeKalb, IL 60115 USA

**Keywords:** Eye tracking, Eye movements, Film perception, Film comprehension, Scene perception, Narrative comprehension, Visual attention, Inferences

## Abstract

Film is ubiquitous, but the processes that guide viewers’ attention while viewing film narratives are poorly understood. In fact, many film theorists and practitioners disagree on whether the film stimulus (bottom-up) or the viewer (top-down) is more important in determining how we watch movies. Reading research has shown a strong connection between eye movements and comprehension, and scene perception studies have shown strong effects of viewing tasks on eye movements, but such idiosyncratic top-down control of gaze in film would be anathema to the universal control mainstream filmmakers typically aim for. Thus, in two experiments we tested whether the eye movements and comprehension relationship similarly held in a classic film example, the famous opening scene of Orson Welles’ *Touch of Evil* (Welles & Zugsmith, Touch of Evil, 1958). Comprehension differences were compared with more volitionally controlled task-based effects on eye movements. To investigate the effects of comprehension on eye movements during film viewing, we manipulated viewers’ comprehension by starting participants at different points in a film, and then tracked their eyes. Overall, the manipulation created large differences in comprehension, but only produced modest differences in eye movements. To amplify top-down effects on eye movements, a task manipulation was designed to prioritize peripheral scene features: a map task. This task manipulation created large differences in eye movements when compared to participants freely viewing the clip for comprehension. Thus, to allow for strong, volitional top-down control of eye movements in film, task manipulations need to make features that are important to narrative comprehension irrelevant to the viewing task. The evidence provided by this experimental case study suggests that filmmakers’ belief in their ability to create systematic gaze behavior across viewers is confirmed, but that this does not indicate universally similar comprehension of the film narrative.

**Electronic supplementary material:**

The online version of this article (doi:10.1186/s41235-017-0080-5) contains supplementary material, which is available to authorized users.

## Significance

Film, television, and video are ubiquitous, and viewers of these media generally have similar narrative experiences despite the complexity of the audiovisual stimuli and large individual differences across viewers. One potential reason for this is the filmmaking techniques for creating highly systematic viewing experiences that filmmakers have intuitively developed and believe to be highly effective. However, these intuitions have rarely been empirically validated. Does film work the way filmmakers think it does? Highly produced mainstream films have been empirically shown to guide viewers to look at the same places at the same time and the association between gaze location and bottom-up visual salience has been reliably computationally modeled. But, the contribution of online top-down cognitive factors, such as comprehension and viewing task, that are known to have large effects on eye movements during reading and static scene viewing are poorly understood for films. This is of critical importance, because although where a person looks and their understanding are highly correlated, if a film viewer has little control over where they look this relationship may be weaker. Our study shows that when viewers watch mainstream movies their visual attention is only modestly affected by differences in narrative comprehension. However, conscious control of one’s attention by a task at odds with comprehending the narrative more strongly guides attention. These results further our understanding of how filmmakers’ and viewers’ goals both shape viewer experience.

## Background

“…and all of us go into a kind of lock step where, if we were watching a tennis match, you’d see that perfect synchronicity of heads going left-right, left-right. The same thing in a movie theatre, when the movie is working and the audience is galvanised, almost hypnotised, all watching the same things, all knowing where to look at the exact same time…it’s a wonderful thing. There is nothing greater than that.” (Spielberg, 2013).

“If a million people see my movie, I hope they see a million different movies.” (Tarantino, 1995)

Watching movies and videos is a ubiquitous activity around the world. Such highly produced videos are very complex stimuli. Yet, they are produced by professional filmmakers for broad entertainment audiences using popular techniques believed to ease viewing and comprehension processes (Smith, 2012), and people seem to comprehend them with little difficulty. Nevertheless, to date little research has been done to explain why this may be the case (Smith, Levin, & Cutting, 2012). One stage of the film comprehension process that might be critical to this apparently effortlessness is how the eyes move across the screen. As demonstrated by the Steven Spielberg quote above, filmmakers believe they have the power to make their audience look exactly where they want them to irrespective of who the audience is. This belief in the primacy of the audiovisual stimulus for guiding attention and, assumedly subsequent comprehension, reflects the tone of practical filmmaking guides (Katz, 1991), accounts of the “rules” used in film construction (Bordwell & Thompson, 2001), and filmmakers’ reflections on their craft (Murch, 2001; Reisz & Millar, 1953). The strong assumption that viewers passively receive a film’s meaning, and that individual differences such as age, gender, race, and sexuality do not impact this reception, was also shared by classic film theories including the Structuralist, Auteurist, Formalist, Marxist, and Psychoanalytic theories (see Stam and Miller (1999) for review).

However, these theories and the view of mainstream filmmakers like Spielberg are out of line with currently dominant film theories. Since the 1960s, advances in film theory have mirrored movements in other areas of the arts to increase the prominence of the individual viewer in theorizing about the act of meaning making (i.e., comprehension). Theoretical movements including Cultural Studies (and its specialist foci such as Feminist and Queer Film Theory), Reception Studies, and Cognitive Film Studies (Bordwell & Carroll, 1996) have acknowledged that films do not have a single meaning but can be read differently by different people, depending on their desires, ideology, and social differences (Hall, 1980). According to these newer film theories, the film experience is similar to a discourse in which, while the viewer is usually passively seated, they are cognitively active in selecting, encoding, and constructing their part of the filmic discourse (Tseng & Bateman, 2013). This view is sometimes also shared by filmmakers who push the boundaries of film, such as Quentin Tarantino (see his earlier quote made when discussing his multi-threaded narrative film *Pulp Fiction*, 1994).

The mental activity of viewers is typically inaccessible to film theorists (except through introspection; Brown, 2015), but a key piece of physical evidence that does exist is how viewers move their eyes on the screen. Furthermore, we can assess their film understanding through their verbal responses to questions. To what extent do film viewers’ eye movements and measured film comprehension support either Spielberg’s belief that he has absolute control over viewers’ gaze and comprehension of a film, or contemporary film theories’ and Tarantino’s assertion that each viewer sees and understands a different film? Empirical disciplines, such as cognitive science, can play a role in exploring the insights of filmmakers and in helping to resolve this debate (Bortolussi & Dixon, 2003; Sanford & Emmott, 2012). In doing so, we can develop and refine theories of how we make sense of media and the role of eye movements in that process.

### Eye movements in scenes

While watching a film, viewers typically move their eyes two to five times per second in order to extract information from it, and those eye movements are likely related to viewers’ understanding of the film they are watching (Eisenstein, 1948; Jesionowski, 1982; Murch, 2001; Smith, 2012). Causally, this relationship can go in two directions: attention guiding comprehension, or what is being comprehended guiding attention. There is a long literature on “bottom-up” features that guide attention in scenes (e.g., color, edges, and motion; Itti, 2005; Mital, Smith, Hill, & Henderson, 2010). In film, these bottom-up features have been shown to have such a strong effect on attention that they lead people to look at the same places at the same times, which has been termed “attentional synchrony” in film (Dorr, Martinetz, Gegenfurtner, & Barth, 2010; Smith, 2013; Smith et al., 2012). Similar synchrony in brain activity has been shown using functional magnetic resonance imaginh (fMRI; Hasson et al., 2008; Shepherd, Steckenfinger, Hasson, & Ghazanfar, 2010) and electroencephalography (EEG; Dmochowski, Sajda, Dias, & Parra, 2012). Such bottom-up influences contrast with “top-down” factors such as the viewer’s task, individual differences, preferences, and the viewer’s active mental model of the scene. The current study asks what role top-down comprehension processes play during film viewing. Although hardly any previous research has addressed this question directly (but see Lahnakoski et al., 2014; Loschky, Larson, Magliano, & Smith, 2015), two well-developed lines of research are highly relevant: research on eye movements and reading comprehension and research on eye movements in static and dynamic scenes.

### Top-down and bottom-up effects on eye movements

At a broad level, comprehension processes for narrative content have been studied in the realm of text (McNamara & Magliano, 2009 for review), and readers’ eye movements have been shown to differ based on their comprehension (reviewed by Rayner, 1998). Importantly, such comprehension effects on eye movements occur at both the local and global levels (Rayner & Morris, 1990; Rayner, Raney, & Pollatsek, 1995). Examples of this relationship at the local level are eye movements associated with the processing of anaphoric references (e.g., identifying the character that is referred to with the pronoun “he”), which typically involve sentences that closely occur in a text (Ehrlich & Rayner, 1983), and generating elaborative inferences that are closely associated with the semantic content of *specific* sentences (O’Brien, Shank, Myers, & Rayner, 1988). Examples at the global level include the fact that, as the overall difficulty of a text increases, readers tend to make more eye movements (Rayner, Chace, Slattery, & Ashby, 2006), that information presented ironically produces more regressive eye movements (Kaakinen, Olkoniemi, Kinnari, & Hyönä, 2014), and that reading times get faster as one progresses through a text (in part due to repetitions of concepts; Rayner et al., 1995). Findings such as these are the basis of the “eye-mind hypothesis” (Just & Carpenter, 1980; Reichle, Pollatsek, Fisher, & Rayner, 1998; Reilly & Radach, 2006) that eye movements are driven by online cognitive processes (e.g., fixation is maintained longer for words that need more processing).

At one level, it is reasonable to assume that comprehension processes are similar for text and film (e.g., Magliano, Loschky, Clinton, & Larson, 2013), and as such one would expect a connection between each movie viewer’s comprehension and their eye movements. More specifically, when movie viewers have different information incorporated into their event models for a narrative (Zwaan & Radvansky, 1998), it seems reasonable that they would attend to different aspects of the film stimulus in order to update their event model, as has been shown for reading text (Anderson & Pichert, 1978).

Similar top-down effects on attention are found during scene viewing. When viewing static scenes, eye movements can be affected by volitional and mandatory top-down processes (Baluch & Itti, 2011). Volitional top-down processes are things like the goal or task of the viewer (Henderson, 2007; reviewed in Henderson & Hollingworth, 1998). Mandatory top-down processes are learned biases that guide attention without any intention to do so (Baluch & Itti, 2011). In the lab, mandatory top-down effects are often implicitly trained during complex search tasks (Baluch & Itti, 2010; Chun & Jiang, 1998). Similar processes could also be argued to occur naturally in scene searches in which context and cognitive relevance have been shown to guide visual search (Eckstein, Drescher, & Shimozaki, 2006; Henderson, Malcolm, & Schandl, 2009; Torralba, Oliva, Castelhano, & Henderson, 2006), and generally in the tendency to fixate faces in scenes (Birmingham, Bischof, & Kingstone, 2008) and the speaker in a scene (Coutrot & Guyader, 2014; Ho, Foulsham, & Kingstone, 2015; Vo, Smith, Mital, & Henderson, 2012). The same is true when watching video clips, in which the *where* and *when* of viewer attention on a screen is influenced by both volitional processes such as the goals of the viewer (Henderson et al., 2009) and more mandatory processes such as who is speaking (Coutrot & Guyader, 2014; Ho et al., 2015; Vo et al., 2012). Alternatively, bottom-up features of scenes are also known to have strong effects on visual attention (Itti, 2005; Mital et al., 2010), but may not affect the interpretation of the scene (Latif, Gehmacher, Castelhano, & Munhall, 2014). When watching films, the role of bottom-up features appears to be very strong, such that when viewing highly produced Hollywood film trailers, people tend to look at *the same places at the same times*, known as “attentional synchrony” (Dorr et al., 2010; Hasson et al., 2008; Itti, 2005; Mital et al., 2010). This is very different from static scenes and “natural videos” (i.e., those lacking a narrative or any filmmaking techniques) in which viewers show lower attentional synchrony (i.e., they may look at similar points of interest, but *not* at the same time) (Dorr et al., 2010; Mannan, Ruddock, & Wooding, 1997).

However, there may be differences in how information is extracted across media (Magliano et al., 2013; Magliano, Higgs, & Clinton, in press). For example, Loughlin, Grossnickle, Dinsmore, and Alexander (2015) showed that visual search is prominent in processing art, but that these processes are not central to making sense of text-based narratives. It may be that narrative film has properties that affect eye movements during comprehension in such a way that the nature of the eye–mind connection is different than how it is manifested in text comprehension.

### Film narrative is unique

Differences between the linguistic and visual modalities of narrative representation need to be accounted for when researching comprehension in visual narratives (Magliano et al., 2013). For example, written text is composed of distinct words arranged in lines and paragraphs on a page, and readers typically fixate every content word (noun, verb, adjective, and adverb) in a line, progressing from left to right (in English). In contrast, films are composed of moving images within a frame, but there are no stated rules for how film viewers should watch them, though filmmakers follow numerous conventions in creating them (Smith, 2012). Also, film shots are typically viewed serially from beginning to end, unless a solitary film viewer uses a remote control with pause and rewind functions. This is in contrast with reading in which the reader controls their pace of reading and can vary the amount of time they allocate to processing a piece of information (i.e., fixation/dwell duration) and make regressive eye movements back to previously read words.

Similarly, the highly produced nature of film contains several features that exert strong bottom-up control and increase attentional synchrony (Dorr et al., 2010; Smith, 2013). Importantly, these features are used based on the practical film theory that they guide viewer attention (Eisenstein, 1948; Murch, 2001; Spielberg, 2013). The bottom-up features include motion (Mital et al., 2010), editing (Wang, Freeman, Merriam, Hasson, & Heeger, 2012), and lighting (Cutting, Brunick, DeLong, Iricinschi, & Candan, 2011; Murch, 2001). Additionally, filmmakers often compose highly produced dynamic scenes to include few points of interest, or construct them such that the bottom-up features guide attention to a single point of interest (Cutting, 2015). Compared to highly produced film, the visual features of both static text and static scenes have relatively weak bottom-up features. Potentially due to the weak bottom-up visual features, many studies have shown strong top-down effects on eye movements in text reading (Hyönä & Lorch, 2004; Rayner et al., 1995; Wiley & Rayner, 2000) and static scene viewing (DeAngelus & Pelz, 2009; Yarbus, 1967). All the above differences between films, reading, and other types of scene viewing suggest that a simple analogy between how viewers process each is likely to be wrong.

### Comprehension and eye movements in film

The few studies that have tested top-down effects on eye movements in film have what may appear to be contradictory effects. Lahnakoski et al. (2014) found that giving viewers an explicit task to take a certain perspective (interior decorator or detective) can have a top-down effect on eye movements. Alternatively, to test the same research question as the current study, how comprehension processes affect eye movements, Loschky, Larson, Magliano, and Smith (2015) presented participants with a scene from the James Bond film *Moonraker* (Broccoli & Gilbert, 1979) and had them start viewing the clip earlier (Context condition) or later (No-context condition). They found that participants had large differences in comprehension due to their context condition, but there were relatively weak effects of comprehension on eye movements. The lack of a top-down effect on eye movements despite large comprehension differences was termed the “Tyranny of Film”. Put differently, the Tyranny of Film is the presence of gaze similarity between groups regardless of comprehension differences between viewers, where gaze similarity refers to groups having the same amount of gaze clustering on the same location(s) in the scene (specific details of the gaze similarity analysis are below).

The few eye-movement differences in Loschky et al. (2015) occurred during a single shot of the clip that was essentially a *static* image that allowed participant gaze to explore the image. In other words, the static nature of the scene may have allowed for eye-movement differences similar to those found in previous experiments using static scenes (DeAngelus & Pelz, 2009; Smith & Mital, 2013; Yarbus, 1967). Nonetheless, the lack of eye-movement differences throughout the rest of the film clip were striking given the large effects typically found during static scene viewing, and the effects found for perspective taking (Lahnakoski et al., 2014) and location-based viewing tasks (Smith & Mital, 2013). Taya, Windridge, and Osman (2012) give evidence for when there is a lack of top-down effects during free-viewing. Similarly, Wang et al. (2012) used a scrambling manipulation with narrative film sequences, which is known to reduce narrative comprehension and memory for texts and picture stories (Gernsbacher, Varner, & Faust, 1990; Larson, Wallace, McQuade, Badke, & Loschky, 2011; Thorndyke, 1977), yet Wang et al. (2012) found very few effects on eye movements except looking at the “most important object” immediately after each cut. This raises the critically important question addressed in the current study, namely, why and when is there a general dissociation between eye movements and film *comprehension*, which fails to support the eye–mind hypothesis in film viewing?

### Overview of the present study

Filmmakers and theorists have long debated the degree to which viewers are active in their consumption of film (see Stam & Miller, 1999 for review). Previous empirical work has found that, in highly composed and rapidly edited films, there seem to be minimal opportunities for top-down impact on narrative processing and gaze (Carmi & Itti, 2006; Hasson et al., 2008; Loschky et al., 2015; Mital et al., 2010; Smith & Mital, 2013). Consistent with these previous works, the present study strategically used a “found film” clip that best illustrated the phenomenon of interest and built an experimental paradigm around it. In this case, based on the prior research described above, we wanted a found film that did *not* conform to the features of a typical, highly produced narrative film (Bordwell, 2002) so that we could create a strong test of top-down comprehension effects on eye movements in film.

We developed selection criteria for a clip based both on its bottom-up features and what it afforded in terms of top-down manipulations. First, the clip needed to lack specific bottom-up features that create attentional synchrony, which should therefore enhance the opportunity for top-down processes to differentially guide viewers’ eye movements while watching the clip. Many film sequences show only a single primary object of interest in each shot, which limits the opportunities for attention to be shifted to different screen locations. A film segment with many different things to look at could reduce the degree of attentional synchrony as different people may look at different things in the film frame. Second, each time there is a film cut (i.e., a switch between camera shots) there is a sudden decrease and then increase in attentional synchrony as viewers search for and then find the point of central interest in the new shot (Carmi & Itti, 2006). A film sequence lacking any cuts for long periods of time (i.e., a “long-take”) would remove the “resetting” after each cut (Mital et al., 2010; Wang et al., 2012). We chose one of the most famous long-takes in film history, the opening scene of Orson Welles’ *Touch of Evil* (Welles & Zugsmith, 1958). This long (3 minutes and 12 seconds) single shot depicts events at a Mexico–USA border crossing in the 1950s. Using a combination of deep-focus, wide framing, and a continuous camera movement that takes in much background action, this shot is a much-discussed example of the type of filmic composition film theorist Andre Bazin viewed as the “ideal” way for cinema to capture reality (Bazin, 1967). Bazin stated that by not cutting but instead choosing to depict the action in a single long-take, directors like Orson Welles (here and in *Citizen Kane*, 1941) evoke:

a more active mental attitude on the part of the spectator and a more positive contribution on his part to the action in progress. While analytical montage [i.e., including many cuts and shots] only calls for him to follow his guide, to let his attention follow along smoothly with that of the director who will choose what he should see, here he is called upon to exercise at least a minimum personal choice. It is from his attention and his will that the meaning of the image in part derives. (Bazin, 1967, p. 35–36)

This Bazin quote (from the perspective of film theory) directly supports our prediction that the opening shot of *Touch of Evil* should provide an ideal opportunity to find top-down influences of viewer’s comprehension on their gaze.

To create top-down comprehension effects on gaze, it may also be necessary to require the viewer to acquire information from different regions within the scene. For example, Taya et al. (2012) found that both experts and novices tend to have high gaze similarity while watching a tennis match. One likely reason for this is that regardless of expertise, there was only one primary thing to watch—namely the ball (and, to a lesser extent, the player whose court the ball was in). Similarly, in the Moonraker clip used in Loschky et al. (2015), there was usually only a single primary object of interest in each shot, thus theoretically increasing the degree of attentional synchrony. For the current study, choosing the *Touch of Evil* clip that has multiple objects in the frame that could be relevant to the narrative at any given moment allows for a top-down manipulation that would require viewers to look in different places (Bazin, 1967).

**Fig. 1:**
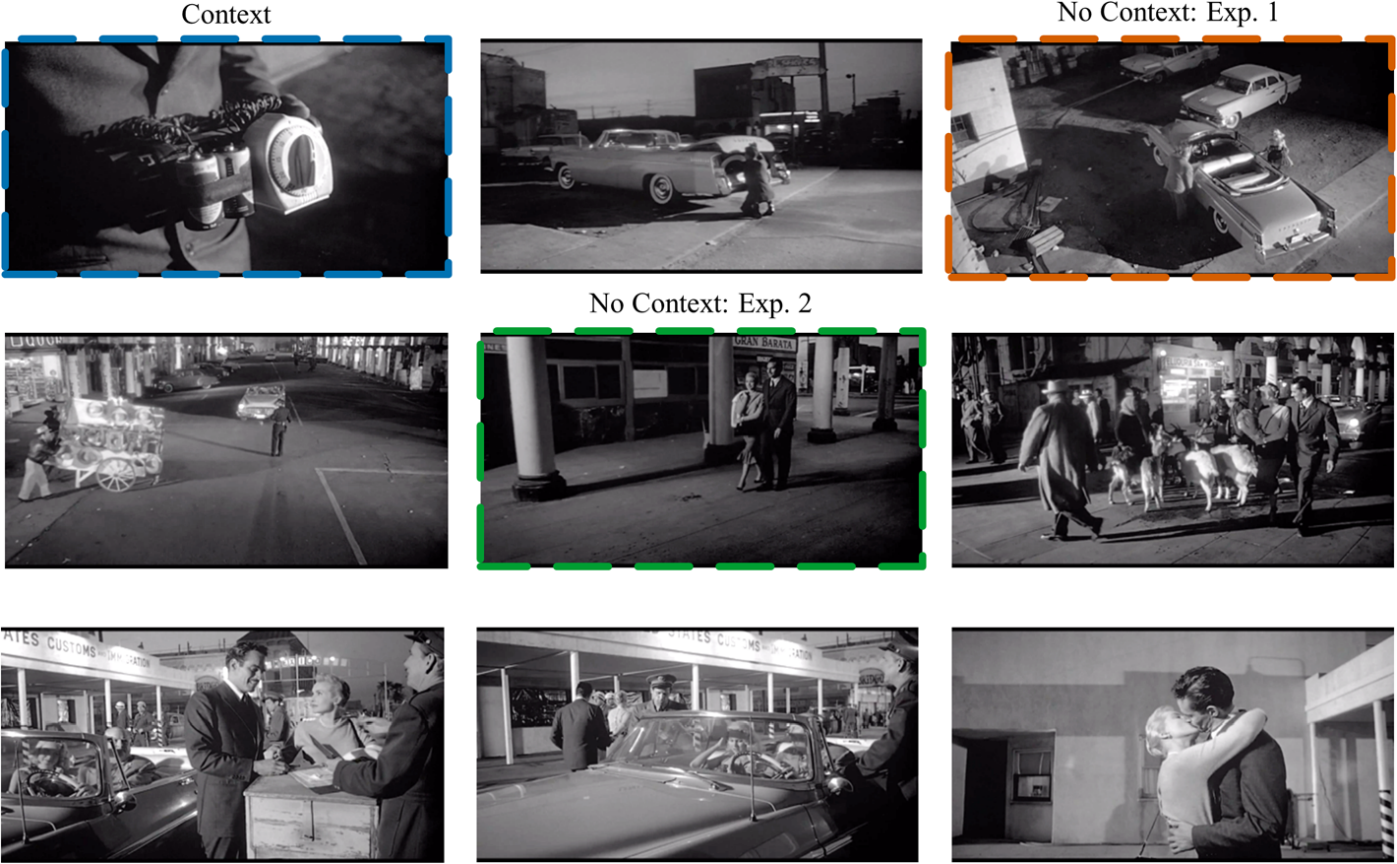
Frames illustrating important shots in the 3 minute 12 second clip from the film *Touch of Evil* (Welles & Zugsmith, 1958). The *blue box* shows the Context condition (experiments 1 and 2), the *orange box* shows the No-context condition (experiment 1), and the *green box* shows the No-context condition (experiment 2)

The narrative content of the opening shot of *Touch of Evil* (https://youtu.be/vIUBoj8CqF8) allows for just such a manipulation of comprehension at the event model level (Kintsch, 1998; van Dijk & Kintsch, 1983). The clip opens on a close-up of someone setting a time bomb (Fig. 1). The time bomb is then placed into the trunk of a car, after which a couple unknowingly gets into the car and drives off, as the camera follows them. About halfway through the clip a second couple walking down the street is introduced (played by Charlton Heston and Janet Leigh, who are mentioned in the quote below), and the camera begins to follow them with the car always lurking around them. Importantly, after the bomb is put into the car, the bomb is never seen again for the remainder of the clip. Many film critics have argued that this creates a very suspenseful experience for viewers as they wait for the time bomb to explode (Comito, 1986; D’Angelo, 2012; Stubbs, 1985), and has been theorized to specifically guide attention to the car:

Our knowledge of the impending explosion makes us hyper-aware of the car’s location, especially in relation to Heston and Leigh (even though we don’t yet know anything about their characters), and Welles expertly teases this instinctive anxiety by allowing it to occasionally leave the frame, getting a few feet ahead of our heroes before being stopped by traffic or passing goats. (D’Angelo, 2012)

The camera does not … move about in order to concentrate and guide our attention. Rather, it seems teasingly to withhold from us what we want to see, what we know--from what we’ve been permitted to see and also from other movies we’ve seen--must be coming. (Comito, 1986, p. 8)

Thus, from a filmmaker’s perspective, it is the knowledge of the bomb that makes the clip so suspenseful—without the bomb, it is just a mundane shot of people and cars on a street. There is theoretical support for these filmmaker intuitions. The presence of the bomb should create a token (i.e., “[bomb]”) in the viewer’s event model that is associated with the car (e.g., Radvansky, 2005), which should be reactivated every time the car is in the frame, including its causal implications for subsequent narrative events (i.e., it creates imminent danger for anyone near it) (e.g., Myers & O’Brien, 1998). Conversely, if a viewer did not see the bomb put in the car, the car would have no particularly salient causal connections in the unfolding narrative, other than as a means of transportation for characters that may or may not be of relevance to the narrative, and would simply be a part of the backgrounded events weakly represented in the event model. Thus, theories of comprehension would say viewers should pay greater attention to the car when the bomb is part of their event model than when it is not. Our comprehension manipulation plays on the power of the bomb to create suspense, and make the car with the bomb an integral component of the scene. Overall, knowledge of the bomb and an impending explosion is at a fairly global level of comprehension, but which gives different levels of importance to local features of the scene (e.g., the car).

To manipulate knowledge of the bomb, we used the “jumped-in-the-middle” paradigm developed by Loschky et al. (2015). This manipulation creates the common experience of coming into a television program or film part way through and then trying to comprehend what is happening. Context group participants saw the bomb being placed in the car at the beginning of the scene, while the No-context group do not see that. At the end of the clip, all participants were asked to predict what would happen next, which provided a basis for demonstrating that the manipulation of context affected comprehension. Eye movements were the primary data of interest.

We assessed both gaze similarity (i.e., the similarity in participant fixation locations on a frame by frame basis) and the extent to which participants fixated on the car with the bomb in the two context conditions. Specifically, the “Event Model” hypothesis (Loschky et al., 2015) predicts there will be greater gaze similarity within conditions (Context and No-context) than between them. This could be the result of, for example, a greater likelihood of viewers fixating on the car in the Context condition than in the No-context condition, as predicted by D’Angelo (2012) above and other film theorists (Comito, 1986; Stubbs, 1985). Overall, Context condition participants looking at the car more would likely result in less exploratory behavior, which would be seen in longer fixation durations and shorter saccades. Alternatively, according to the Tyranny of Film hypothesis (Loschky et al., 2015), the attentional synchrony created by the bottom-up features of this masterfully produced film (created by virtuoso filmmaker Orson Welles) would limit the impact of top-down comprehension on eye movements. Specifically, if everyone is looking at the same places at the same times, there would be no room for differences in eye movements due to differences in viewers’ comprehension. As such, this hypothesis predicts comparable levels of gaze similarity and number of fixations on the car with the bomb in the two context conditions. Importantly, in comparison to the James Bond *Moonraker* clip used in Loschky et al. (2015), the comparatively weaker bottom-up features of the *Touch of Evil* clip should theoretically reduce the attentional synchrony. Nevertheless, it is conceivable that Welles used all the other filmmaking techniques at his disposal (mis-en-scene [i.e., staging], camera framing [i.e., what is shown on camera], camera movement, etc.) to masterfully guide viewers’ attention.

This found film approach is valuable in situations where it is difficult to equate stimuli on the features of interest, which is the case when using naturalistic films that vary dramatically in visual cinematic features that can affect attention and eye movements. Additionally, it is intended to demonstrate a phenomenon that already exists in the world that should subsequently be studied in a more controlled manner, likely with multiple experimenter-generated video clips. Importantly, the present study was carried out as a replication and extension to Loschky et al. (2015), thus adding greater generalizability to it, and providing new and deeper insights.

## Experiment 1: context and eye movements

### Methods

Experiment 1 tested the effect of the comprehension differences on viewers’ eye movements while watching the film clip.

#### Participants

Eighty-four participants (61 females; mean age = 18.6 years; standard deviation (SD) = 1.4) were pseudo-randomly assigned to one of two viewing conditions for the opening scene of *Touch of Evil* (Context, n = 42; No-context, n = 42). The Kansas State University Institutional Review Board approved all experiments in the study. The study was determined to pose minimal risk to the participants and informed consent was deemed unnecessary (i.e., exempt under the criteria set forth in the Federal Policy for the Protection of Human Subjects.) All participants received course credit for their participation, and all analyses were performed on de-identified data.

#### Stimuli

Two clips from the opening scene of Orson Welles *Touch of Evil* were used (Welles & Zugsmith, 1958). The Context version shows a bomb being placed in a car trunk at the beginning and runs for 3:12. The No-context version omits the first 18 seconds when the bomb is placed in the car and runs for 2:54. Both clips end with a close-up of the walking couple kissing. An initial experiment (Hutson, Magliano, Smith, & Loschky, Working memory span and film comprehension: Effects on high-level inference generation, in preparation, not presented here, found that presenting the clip with audio created the largest effect between the Context and No-context conditions in inference generation. Thus, audio was presented with the film clip.

Both clips were presented at a frame rate of 30 frames per second (fps) and a resolution of 1080 × 720 pixels. The video clips were shown on a 17” ViewSonic Graphics Series CRT monitor (Model G90fb). A chin and forehead rest set a fixed viewing distance of 60.96 cm. The screen subtended 21.42° × 16.10° of visual angle.

Eye tracking was done using an EyeLink1000 eye tracker (SR Research), which samples eye position 1000 times per second (1000 Hz). Based on the SR Research guidelines, an average spatial accuracy of 0.5° of visual angle and a maximum error of 1° or better were obtained for all calibrations.^1^

#### Procedure

All participants were told that they would be shown a video clip while their eyes were tracked. Participants went through a nine-point calibration routine, after which the experiment began. An eye-movement trigger was used to ensure that the video started at the beginning of a fixation. To start a trial, while the participant was looking at the central fixation point, they pressed a button which moved the fixation point 13.65° to right of center. Once the participant fixated the new point, it moved back to the center. During the saccade (velocity > 30°/s) back to the center, the video began to play. In this way, any saccadic inhibition (which increases the current fixation duration), caused by the motion transient due to the sudden onset of the video clip, was masked by the viewer’s own eye movement (Reingold & Stampe, 2000, 2002). Participants then watched the video, uninterrupted, until the moment when the couple kisses (3:12 into the Context condition and 2:54 into the No-context condition). At the end of the video all participants were asked, “What will happen next?” and responses were collected using the computer keyboard. The next question asked was, “Have you seen this movie before?” The keyboard was used to indicate “Yes” or “No.” If a participant responded “Yes” they were asked the follow-up question, “What was the name of the movie?” No participants indicated having seen the movie before.

#### Data analysis

To identify whether participants’ predictive inferences at the end of the clip were influenced by having the bomb in their event model, we had two research assistants code each inference, with coders blind to the condition from which each response was taken. The coding of the inference was dichotomous from (1 = participant mentioned something related to the bomb, 0 = the participant did not). The coders had a high level of inter-rater reliability (Cohen’s *Kappa* = 0.954, *p* < 0.001). Any remaining discrepancies between the two coders were resolved through discussion. After coding, the four participant groups were Context + Inference (*n* = 33), Context + No-inference (*n* = 9), No-context + Inference (*n* = 1), and No-context + No-inference (*n* = 41).

In this and all following experiments, Bayes factors (BF_01_ reported) (Rouder, Morey, Speckman, & Province, 2012; Rouder, Speckman, Sun, Morey, & Iverson, 2009; Wetzels & Wagenmakers, 2012) were calculated for tests that did not reject the null to identify the level of evidence for the null, which would support the Tyranny of Film hypothesis. Values over 1 offer some evidence for the null, over 3 is substantial evidence, and over 10 is strong evidence for the null.

An important consideration when analyzing eye-movement data in videos is that there may be smooth pursuit eye movements, which are low-velocity eye movements during which visual information still reaches the visual cortex. Unfortunately, there are still no reliable methods for parsing eye-movement data to differentiate between fixations and smooth pursuits (Larsson, Nyström, Ardö, Åström, & Stridh, 2016). Nevertheless, this issue was addressed by rerunning analyses that didn’t already account for smooth pursuit with a cleaning procedure to remove potential smooth pursuits. The cleaning procedure quantified the maximum linear displacements during intersaccadic intervals (the period when the eye-tracker eye-movement parser estimated that the eyes were in a fixation due to their velocity being lower than the saccade threshold). The change in eye location during the intersaccadic interval was identified first by calculating the Euclidean distance in pixels between the x,y location of where the saccade before the intersaccadic interval ended and the location of where the next saccade began. This pixel value was then converted to the degrees of visual angle that the eyes moved during each intersaccadic interval. The majority of these intersaccadic intervals are likely to be fixations and exhibit low displacement. However, potential smooth pursuits, by definition, require displacement along with a moving target and can therefore be excluded by removing all intersaccadic intervals with displacements greater than 1° of visual angle. Surprisingly, this resulted in about 30% of all previously identified fixations being cleaned from the data set, regardless of experiment or condition. This is a noticeably higher proportion of potential pursuits than has previously been reported for video viewing (e.g., 2.8% of all data; Smith & Mital, 2013). However, despite the large number of previously identified fixations being removed from the data, the effects found for the remaining fixations (with low displacement; unlikely to be pursuit periods) were unchanged. Below, we report results both with and without the intrasaccadic cleaning to remove potential smooth pursuit eye movements from fixations.

### Results

#### Overview

As will be described in detail below, the results of experiment 1 showed that although there were large differences in participant comprehension based on the context manipulation, the only eye-movement effect showed Context participants who made the inference had longer saccade lengths than No-context participants. Bayes factors indicated that all other effects (fixation durations, gaze similarity, and region of interest) supported the null hypothesis. Thus, experiment 1 mostly supported the Tyranny of Film hypothesis.

#### Predictive inference

A chi-square test was used to identify whether there was a comprehension difference between the Context and No-context conditions. The expected difference between context conditions was found, with 80% of participants in the Context condition making a bomb-relevant inference compared to only one participant from the No-context condition doing so (*X* ^*2*^ (1, *N* = 85) = 51.59, *p* < 0.001). There were also qualitative differences in the predictive inferences generated. Specifically, instead of predicting that the bomb would explode, killing the couple in the car, other innocent bystanders, and possibly the walking couple, a common predictive inference among those in the No-context condition was that the couple in the car would have dinner with the walking couple. Thus, the results indicate viewers in the two conditions had radically different event models (i.e., comprehension) of the narrative in the film clip based on the context they were given.^2^

#### Eye movements

##### Fixation durations and saccade lengths

Fixation durations and saccade lengths can be very sensitive to manipulations of comprehension in reading at both local and global levels (Rayner, 1998), what is currently being fixated in scenes (Henderson & Pierce, 2008; Henderson & Smith, 2009), and manipulations of task in dynamic scenes (Smith & Mital, 2013). The event model hypothesis thus predicts there should be effects of our comprehension manipulation on these basic eye-movement metrics in the current study. Specifically, based on the logic that Context condition participants will hold knowledge of the bomb in their event model, the Event Model hypothesis would predict that when the car with the bomb is on the screen they should have tighter gaze on the car. This should result in shorter saccades and longer fixations. The inclusion of these measures should give a fuller picture of the eye-movement results to help interpret the effects for gaze similarity and region of interest below.

All eye-movement data were first cleaned by removing the longest and shortest 1% of fixation durations and saccade lengths for each participant. We then compared the mean fixation durations and saccade lengths between the Context and No-context groups for the shared viewing period. There were no significant differences in fixation duration between the two conditions. In the Context condition, the average fixation duration was slightly descriptively longer than the No-context condition (Table 1), but not significantly different (*t* (82) = 0.438, *p* = 0.662; intersaccadic interval 1° cleaning, *t* (82) = 0.888, *p* = 0.377). There was substantial evidence for the null hypothesis (BF_01_ = 5.48). The effect was the same when only participants in the Context condition who made the inference were compared to the No-context condition (*t* (73) = 0.318, *p* = 0.751; intersaccadic cleaning, *t* (73) = 0.772, *p* = 0.443). There was again substantial evidence for the null hypothesis (BF_01_ = 5.33). The average saccade length for the Context group was descriptively longer than for the No-context group, and marginally significant (*t* (82) = 1.848, *p* = 0.068, *d* = 0.41; intersaccadic cleaning, *t* (82) = 1.892, *p* = 0.062). The Bayes factor only showed anecdotal evidence for the null hypothesis (BF_01_ = 1.25). When only Context condition participants who made the inference were included the effect of condition and inference on saccade lengths was significant (*t* (73) = 2.089, *p* = 0.040; intersaccadic cleaning, *t* (73) = 0.2.168, *p* = 0.033). Thus, Table 1 shows participants in the context condition *who made the inference* had longer saccade lengths compared to the No-context condition, though this was a small-to-medium effect (*d* = 0.489).

**Table 1:** Experiment 1 and 2 fixation duration and saccade length descriptive statistics

Experiment	Condition	Mean	SD
Fixation durations		Milliseconds	
Experiment 1	Context (inference)	391	68
	No-context	386	63
Experiment 2a	Context (inference)	367	57
	Context (no-inference)	378	70
	No-context	398	82
Experiment 2b	Map task	361	47
Saccade lengths		Degrees	
Experiment 1	Context (inference)	4.89	.58
	No-context	4.63	.66
Experiment 2a	Context (inference)	4.89	.61
	Context (no-inference)	4.65	.74
	No-context	4.69	.82
Experiment 2b	Map task	5.39	.68

The descriptive statistics are only presented for the main analyses run. The analyses run with different cleaning methods did change the mean values for fixation durations and saccade lengths, but since the interpretations did not change based on the inferential statistics those means are not presented

Longer saccade lengths usually show greater exploration of a scene (Pannasch, Helmert, Roth, Herbold, & Walter, 2008; Smith & Mital, 2013), which makes it surprising the Context group that made the inference would explore more. They have the best understanding of the narrative presented, and many of them maintained the bomb in their event model throughout the clip. This should create suspense that would guide their eye movements towards the car with the bomb, which should theoretically result in shorter saccade lengths. Bezdek et al. (2015) showed in an fMRI study that suspense in film narrows attentional focus. A potential alternative explanation is that the Context participants who made the inference did explore the scene more to look for potential effects of a bomb explosion. Also, there is the possibility that, due to their relatively good comprehension for the narrative, Context participants that made the inference were under less cognitive load to maintain the narrative, which gave them the opportunity to explore the screen more. This may be similar to a person watching a film for the second or third time, and noticing things they hadn’t in previous viewings because they don’t have to follow the narrative as closely. Nevertheless, this difference in saccade lengths was a relatively small effect, so the above interpretations must be made cautiously.

#### Gaze similarity

##### Data pre-processing

Comparing the spatiotemporal distribution of gaze between viewing conditions in dynamic media is more difficult than in static scenes as traditional scanpath comparison methods assume that the stimulus does not change during viewing (e.g., Scanmatch; Cristino, Mathot, Theeuwes & Gilchrist, 2010). Instead, a method based on comparison of gaze heatmaps between conditions on a frame-by-frame basis can be used. (Note that, for this reason, it is not necessary to clean out potential smooth pursuit eye movements, since the analysis simply calculates each viewer’s mean gaze position during that 1/30th of a second [i.e., 33 ms].) Gaze heatmaps represent the probabilistic spatial distribution of raw gaze points within a viewing condition and can be compared across conditions to statistically confirm qualitatively observable changes in heatmaps over time (e.g., tightening of gaze clusters = moments of *attentional* synchrony) and differences between heatmaps (i.e., when gaze similarity is high or low). Such heatmap comparison methods have become the standard in dynamic scene viewing research (Peters, Iyer, Itti, & Koch, 2005; Dorr et al., 2010; Caldara & Miellet, 2011; Loschky et al., 2015). The method used here is an adaptation of the Normalized Scanpath Saliency (NSS) first proposed by Peters et al. (2005) and extended to video by Dorr et al. (2010) (for full details of the method and equations, see Dorr et al. (2010) and Loschky et al. (2015)). Our gaze similarity derivation of NSS is preferable over alternative methods of comparing the distribution of gaze between two groups. For example, the alternative of averaging the separate Pearson correlations of gaze X and Y coordinates would ignore the 2D nature of the data at a particular moment, and also produce similarly high correlations whether each distribution is tightly clustered or not. The gaze similarity measure also allows us to use inferential statistics to identify moments when the gaze distributions between two groups differ in time, allowing for direct tests of the Event Model hypothesis*.*

The NSS method was modified for the analysis here in two critical ways. First, to calculate inter-observer similarity *within* the reference condition (in this case, the Context condition since it is the originally intended viewing condition by the filmmaker), a probability map is created by down-sampling the raw eye-tracking data to 33 Hz (from 1000 Hz) to express raw eye fixation X/Y coordinates per video frame, and exclude saccades and blinks (periods when visual encoding is absent). A 2D circular Gaussian (1.2° SD; roughly equivalent to the fovea) is then plotted around each raw gaze location and temporally averaged over a 225-ms moving time window (to roughly approximate the duration of an average fixation) for all but one participant within the Context condition. These Gaussians are summed and normalized relative to the mean and SD of these values across the entire Context condition, to see how the similarity fluctuates over time (z-score similarity = (Raw values − Mean)/SD). The gaze location of the remaining participant is then sampled from this distribution (i.e., a z-score is calculated for this participant) to identify how their gaze fits within the distribution at that moment. This “leave-one-out” procedure is repeated for all participants within the Context condition until each participant has a z-scored value (referred to as “gaze similarity” here). These values express both 1) how each individual gaze location fits within the group at that moment, and 2) how the average gaze similarity across all participants at that moment differs from other times in the video: a z-score close to zero indicates average synchrony, negative values indicate less synchrony than the mean (i.e., more variance), and positive values indicate more synchrony.

**Fig. 2:**
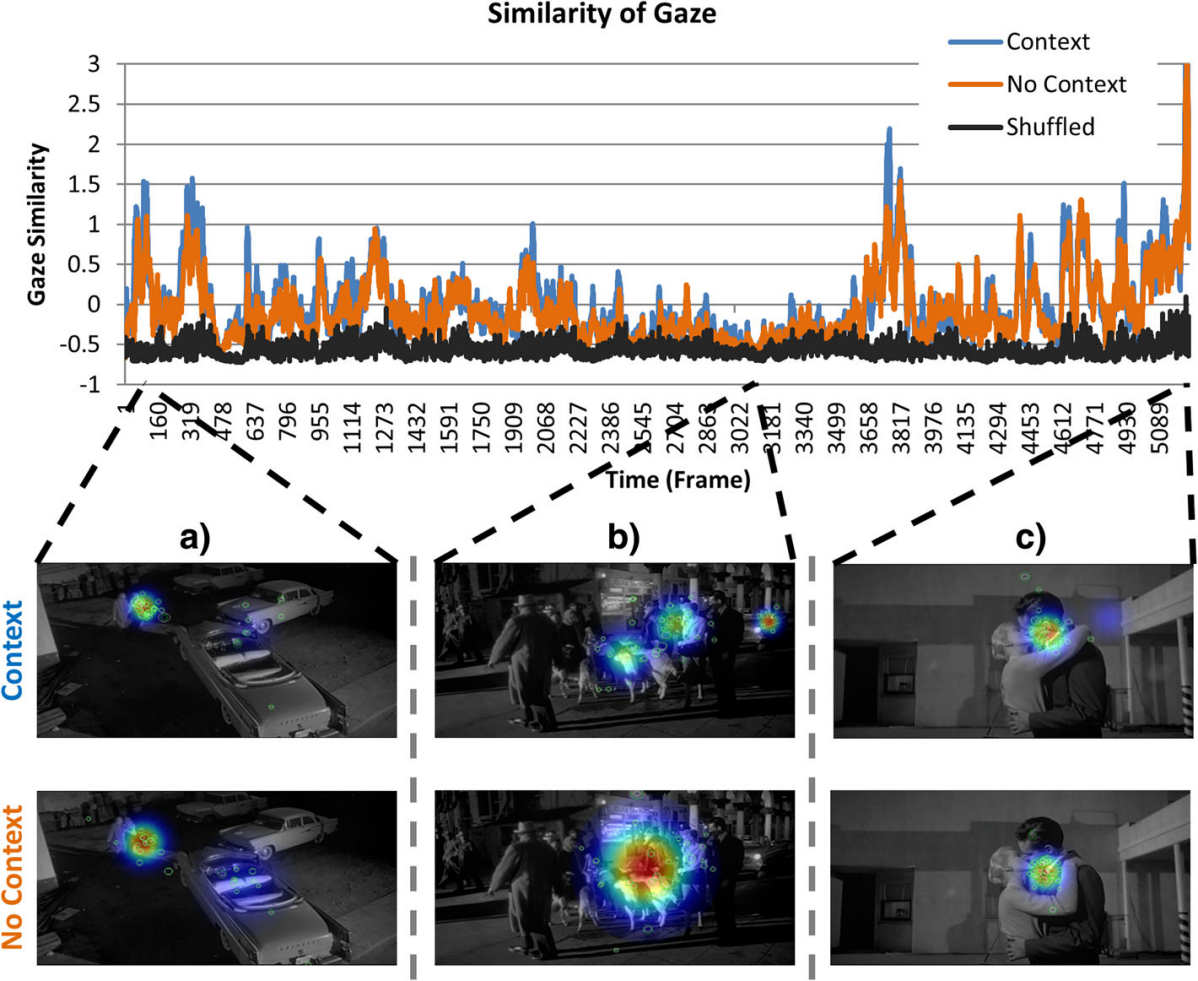
*Top*: Similarity of gaze by context condition across the shared viewing period of the clip. Gaze similarity is expressed as a z-score probability relative to the Context condition. (Context [*blue*], No-context [*orange*], and shuffled baseline [*black*]). Large values indicate greater gaze similarity. *Bottom*: Three of the peaks in gaze similarity are illustrated by image frames with superimposed heat maps of participant gaze location. The frames show the gaze heat maps at the points indicated on the gaze similarity figure for both the Context and No-context conditions. Frames **a** and **c** show high gaze similarity, while frame **b** shows low gaze similarity. Note that frame **c** was the single highest level of attentional synchrony in the entire film clip. Gaze heatmaps produced by CARPE (Computational and Algorithmic Representation and Processing of Eye-movements; Mital et al., 2010)

Second, the method is extended to allow gaze from different viewing conditions (e.g., No-context) to be sampled from a reference distribution (Context). For each gaze point in the No-context condition, the probability that it belongs to the Context condition’s distribution is identified by sampling the value at that location from the Context’s probability distribution (this time leave-one-out is not used as the gaze does not belong to the same distribution so cannot be sampled twice). The resulting raw NSS values for No-context are then normalized to the reference condition. Importantly, if the two distributions are identical, the average z-scored similarity for both distributions will fluctuate together, expressing more (positive z-score) or less (negative z-score) gaze similarity together over time (Fig. 2). However, as the similarity score is derived from the reference distribution, if the two distributions differ significantly, we cannot know if this is because the comparison distribution has more versus less gaze clustering than the reference distribution. We can only say that the comparison distribution differs more from the reference distribution than the reference distribution differs from itself. For example, both gaze distributions could be tightly clustered but in different, non-overlapping parts of the screen or the comparison distribution could be more spread out and only partly overlap with the tight reference distribution. Both situations would result in significant differences between the distributions.

To address this ambiguity of the gaze similarity measure, we also include three other gaze measures in our analyses. Specifically, we include two common measures of attentional synchrony, sum-weighted gaze covariance and number of gaze clusters (Mital et al., 2010), to determine the degree to which gaze in each group was tightly clustered (We report these later for all experiments and groups in Fig. 8.) We also report region of interest analyses to determine the degree to which gaze goes to specific regions in each condition. By including all four gaze measures (gaze similarity, sum-weighted gaze covariance, number of gaze clusters, and the car region of interest analysis), we can disambiguate the sources of differences in gaze similarity.

Additionally, a shuffled baseline was created to demonstrate the gaze similarity that would be observed if eye movements were randomly distributed during the clip (given the constraints of normal eye movements within that clip). The shuffled baseline started with the Context group. Because the gaze similarity values are calculated based on each participant’s gaze location on each film frame, the order of frames for each participant (and thus the order of their eye movements across frames) was shuffled. In other words, for the first frame of the film, instead of having each participant’s first fixation the shuffled baseline may have one participant’s first fixation, a second participant’s 356th fixation, and another’s 22nd, etc. This new gaze distribution was then compared to the reference distribution (i.e., Context) in the same method described above. This created a chance baseline of gaze similarity which represents a floor value of similarity for each participant at every frame against which all other conditions can be compared. Importantly, since the shuffled baseline was created from eye movements from the same participants in the same clip, any biases within the clip (e.g., center bias or certain characters staying on a specific side of the scene) would be in the baseline as well. These frame-by-frame gaze similarity z-scores for each participant and condition (including the shuffled baseline) were then analyzed to identify differences in gaze similarity across conditions.

##### Gaze similarity results

As shown in Fig. 2, the first gaze similarity analysis compared the Context and No-context conditions across the entirety of the shared viewing period of the film clip that overlapped across conditions (i.e., the 2 minutes and 54 seconds of film, starting just after the bomb was placed in the car).

Qualitatively, looking at Fig. 2 one can see that gaze similarity scores between the Context and No-context groups generally overlap throughout the film clip, indicating that, regardless of context condition, viewers likely did not differ in their overall gaze similarity. A *t*-test of mean gaze similarity between groups averaged across all frames supported this qualitative assessment (*t* (80) = 1.081, *p* = 0.283; *d* = 0.241), indicating that knowledge of the bomb did not have an effect on overall viewer gaze similarity. Additionally, the Bayes factor showed substantial evidence for the null (BF_01_ = 3.45), namely support for the Tyranny of Film hypothesis. The results are similar when participants who did not make the inference in the Context condition are removed from the analysis (*t* (71) = 0.592, *p* = 0.556). Next, we included the shuffled baseline for comparison. As shown in Fig. 2, when the experimental groups’ gaze similarity is above the shuffled baseline, it indicates that gaze is more clustered than would be predicted by chance, possibly due to either the bottom-up features of the film or all viewers’ mental models systematically guiding their eye movements. This qualitative assessment was confirmed by adding the shuffled baseline to the ANOVA for condition, which produced a significant effect (*F* (2, 122) = 73.727, *p* < 0.001, *ηp2* = 0.551). Bonferroni corrected pairwise comparisons indicated that both the Context (mean (M) = −0.001, SD = 0.267) and No-context (M = −0.067, SD = 0.290) conditions had significantly greater gaze similarity than the shuffled baseline (M = −0.561, SD = 0.034). Importantly, Fig. 2 shows that this quantitative difference in gaze similarity from chance was highly systematic. For example, Fig. 2b shows the time point with the lowest gaze similarity, which shows a busy street scene with many people, goats, cars, and building signs to look at. Conversely, Fig. 2c shows the moment of highest gaze similarity, which is when the walking couple kiss, at the center of the screen, and there is nothing else of interest to look at (i.e., only a non-descript architectural background). Therefore, one cannot attribute the null effect of context on gaze similarity to the gaze similarity measure being insensitive to variations in attentional synchrony. On the contrary, Fig. 2, and comparisons with the shuffled baseline, shows that the gaze similarity measure was very sensitive to moments when one would predict to find lesser (Fig. 2b) or greater (Fig. 2c) attentional synchrony.

#### Region of interest

##### Data pre-processing

Dynamic regions of interest were created for the clip to test whether either condition looked more at the car with the bomb in it. To create the dynamic region of interest for the car, we used Gazeatron (Vo et al., 2012) to identify the rectangular x,y pixel coordinates for the car on the screen for each frame (at 30 frames/s). These pixel coordinates were then exported and combined with the raw fixation report from EyeLink DataViewer (SR Research). This was used to calculate the cumulative dwell time and mean number of fixations for each participant in the car region of interest. One-second time bins were used, and fixations were counted for the time bin they ended in (i.e., if a fixation went across time bins, it was only counted for the time bin it was in when the next saccade was generated).

##### Region of interest results

While gaze similarity is a metric that indicates the co-occurrence of eye movements in space and time, it does not indicate the features of a scene that are being attended to. The region of interest analysis remedies this by indicating how much a specific object in a scene, here the car with the bomb in it, is attended to. The Event Model hypothesis predicted that the car with the bomb would be of greater importance to participants in the Context condition, because they are aware of the potential destructive causal effects the car could have on nearby persons, places, and things.

**Fig. 3:**
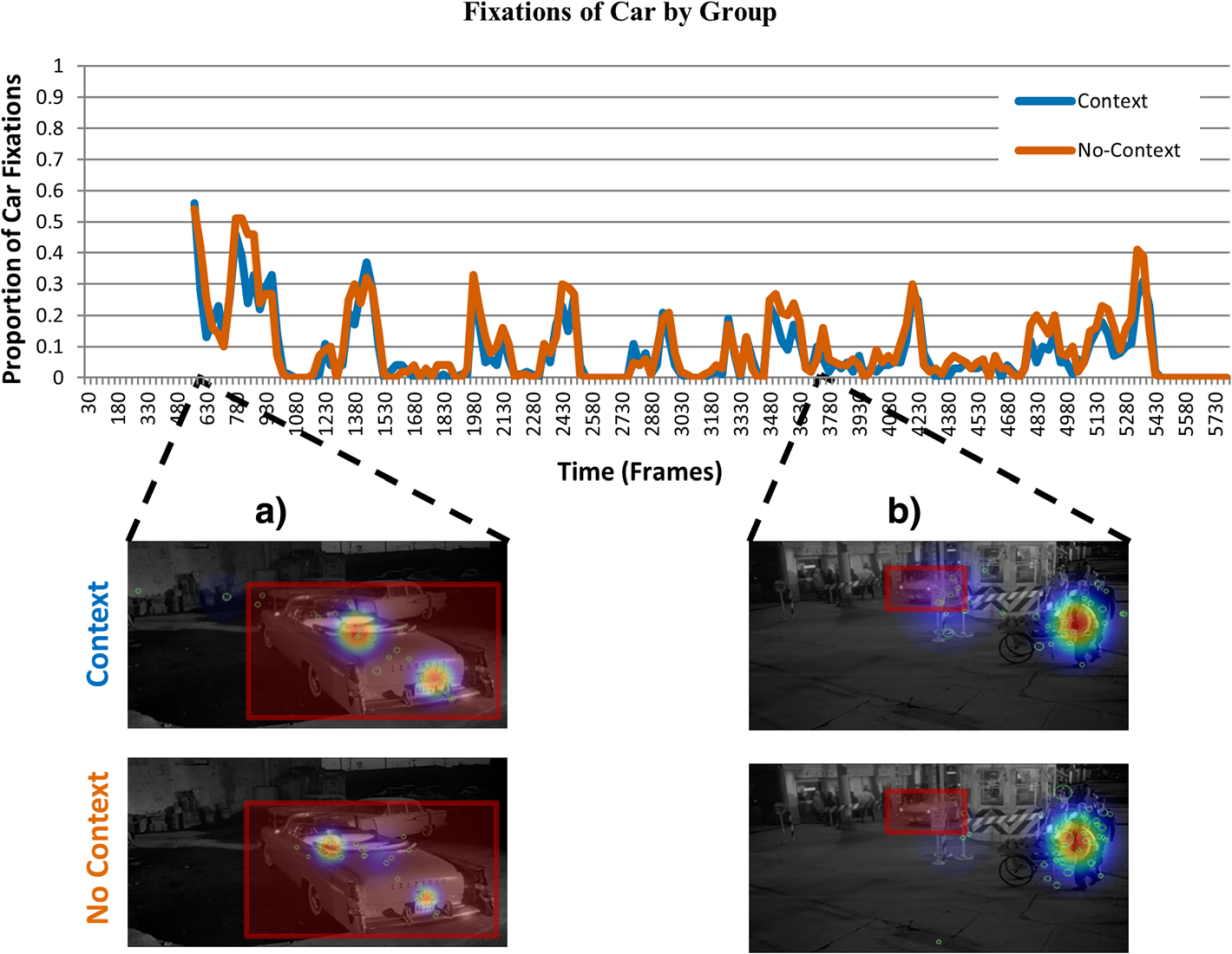
*Top*: Proportion of participants fixating the car by context condition throughout the film clip (shown here in 3-s bins due to resolution limitations of the figure). The higher the value, the more participants were looking at the car. *Bottom*: Film stills show the region of interest for the frame, with fixation and heat maps superimposed. Still set **a** shows a time point when the car was fixated by most participants, and set **b** shows when the car was minimally fixated. Note that frame **a** shows the single highest proportion of viewers fixating the car region of interest, which was at the start of the common viewing period across both context conditions

As illustrated in Fig. 3, as with the gaze similarity analysis, fixations on the car by viewers in the two context conditions were compared for the shared viewing time from the start time of the No-context condition when both conditions were seeing the exact same information. The region of interest was used to calculate the mean number of fixations when the car was present on the screen within 30-frame (1 s) time bins.

As with the gaze similarity analyses, Fig. 3 shows the lines for the two context conditions are mostly overlapping, indicating that, regardless of the context condition, participants fixated the car at the same time points throughout the clip. A *t*-test comparing the proportion of fixations on the car between each condition was not statistically significant (*t* (82) = 1.73, *p* = 0.087; *d* = 0.382), and the Bayes factor showed anecdotal evidence for the null, namely the Tyranny of Film hypothesis (BF_01_ = 1.52). The non-significant trend was for the No-context condition to have a higher proportion of fixations (M = 0.098, SD = 0.045) on the car than the Context group (M = 0.082, SD = 0.036), which is in the opposite direction of what was predicted. The result was the same when only those members of the Context condition who made the inference were included (*t* (73) = 1.434, *p* = 0.156; *d* = 0.316), which also showed anecdotal evidence for the null (BF_01_ = 2.21). Overall, this indicates that the viewers without knowledge of the bomb fixated the car at a similar rate to those with knowledge of it.

### Discussion

The context manipulation had an impact on comprehension in that participants in the Context condition were more likely to predict that the car would explode than those in the No-context condition. However, context had only one modest effect on eye movements. Saccade lengths were slightly longer for participants in the Context condition that made the inference about the bomb. This could potentially be argued to support the hypothesis that Context participants explored the scene more. However, all other eye-movement measures showed no effect. This included gaze similarity, which should pick up on one condition exploring the scene more than the other. These results therefore mostly support the Tyranny of Film hypothesis (Loschky et al., 2015). This is despite the fact that, as shown in Fig. 8, the *Touch of Evil* film clip produced less gaze clustering than the *Moonraker* clip (Loschky et al., 2015), which we had predicted due to the *Touch of Evil* clip lacking cuts but including numerous objects in the film frame to look at. This stands in contrast to the lack of effect of strong comprehension differences on viewers’ eye movements. Thus, our predicted reduction of gaze similarity in the *Touch of Evil* film clip in comparison to the *Moonraker* film clip was nevertheless not enough to allow a strong difference in comprehension between context conditions to produce meaningful differences in eye movements.

One potential problem with experiment 1 was that the No-context condition and the Context condition both show the car at the beginning of the scene, and in particular a couple getting into the car. First mentioned entities have a special status in event models for text narratives (e.g., Gernsbacher, 1990). As such, the car was likely prominent in the event models for both the Context and No-context conditions, which may have led to similar eye movements in both conditions, regardless of whether they had knowledge of the bomb.

## Experiments 2a and 2b: eye-tracking with new No-context condition and map task

In order to address the potential first mention (Gernsbacher, 1990) issue that viewers in the No-context condition may have treated the characters in the car as protagonists, and therefore paid close attention to the car even though they did not know it contained a bomb, a new No-context condition was used. In the new No-context condition, viewers began watching the clip only after the walking couple entered the street and the car was off-screen (Fig. 1; image 5 marked “No-context: Exp. 2”). Thus, viewers in the new No-context condition should treat the walking couple as the protagonists. Viewers in the Context condition started watching the clip from the beginning as before.

Experiment 2b was conducted to demonstrate that the Tyranny of Film effect can be broken when there are strong endogenous factors affecting attention. Previous research has shown higher level cognition has large online effects on eye movements during scene viewing (Foulsham & Underwood, 2007; Henderson, Brockmole, Castelhano, & Mack, 2007; Henderson, Shinkareva, Wang, Luke, & Olejarczyk, 2013). These effects have also been shown more recently in “natural film” (i.e., unedited real-world video), such as trying to determine the location depicted in a video (Smith & Mital, 2013), and in edited narrative film, such as taking different film viewing perspectives (Lahnakoski et al., 2014). We therefore predicted viewer eye movements in the *Touch of Evil* clip would similarly be affected by a cognitive task that was designed to be specifically at odds with understanding the narrative, specifically a map drawing task that involved creating a map of the narrative spatial environment. That is, eye movements for viewers under instruction to draw a map would be different than those of participants processing the film clip in order to comprehend it. Moreover, those eye movements under the map task would be at regions of the screen important to the task, but not important to the narrative content.

### Methods

#### Participants

For experiment 2a, data were collected from 201 students enrolled in an introductory psychology course at Kansas State University for course research credit. Data from eight participants were dropped because of program errors during data collection, for not completing the questions at the end of the experiment, or for having participated in an earlier experiment using *Touch of Evil*. Data from the remaining 193 participants (age M = 19.5 years, female = 59.2%) were included in the analyses. Participants were pseudo-randomly assigned to either the Context condition (n = 131) or the new No-context condition (n = 62 participants) with the constraint that we have roughly twice as many participants in the Context condition, based on the assumption (from experiment 1 and further comprehension experiments (Hutson et al., Working memory span and film comprehension: Effects on high-level inference generation, in preparation)) that roughly 50% of them would fail to generate a bomb-relevant predictive inference at the end of the film clip. Based on this assumption, we expected to end up with three roughly equally sized groups of participants: Context condition + Inference, Context condition + No-inference, and No-context condition + No-inference.

For experiment 2b data were collected from an additional 75 participants. For this additional experimental condition, a priori criteria were created to select which participants would be included in analyses based on performance on a map drawing task described below. First, the Gardony Map Analyzer was used to score participants’ maps for accuracy (Gardony, Taylor, & Brunyé, 2015), and only participants with scores greater than or equal to the median were included in any of the analyses (see “Data analysis” section for specifics on the Gardony Map Analyzer and Additional file 1: Appendix B for example maps with scores). Second, only participants that *did not* make the inference about the bomb at the end of the clip were included in the eye-movement analyses. This is because one purpose of the map task was to make the narrative irrelevant, and participants able to make the inference about the bomb must have attended to the narrative. This resulted in a total of 37 comparison group participants being included in eye-movement data analyses. Future analyses may look at more fine-grained comparisons of all of these participants using map scores as a continuous predictor of eye-movement variability.

#### Stimuli

The same opening scene from *Touch of Evil* was used in experiment 2, but no audio was presented with the clip. This decision was made because the goal of this research is to explore how narrative comprehension affects eye movements, and we became aware that the film included dialogue in the last 15 seconds of the clip to subtly remind viewers of the bomb.^3^ Since the dialogue occurred at the end of the clip, it could not have an effect on the majority of the eye movements in the majority of the clip. However, removing the subtle reminder of the bomb allows us to make a cleaner comparison of the eye movements of those viewers in the Context condition who did versus did not mention the bomb in their predictive inferences. The clip used for the Context condition was otherwise identical to that used in experiment 1. The new No-context condition saw a different version of the clip that started 1 minute and 49 seconds into the opening scene, at a point when the walking couple was shown alone on the screen, with the car off-screen. The same display set up from experiment 1 was used in experiments 2a and 2b.

#### Procedure

Experiment 2a procedures were identical to those of experiment 1. With respect to experiment 2b, all stimuli and procedures were identical to the Context condition in experiment 2a except for the inclusion of the map task. Specifically, all participants were presented the Context version of the film clip. However, before presentation of the clip, participants were given the map task instructions to draw a detailed map of the locations in the scene, including labels, at the end of the clip from memory (full instructions in Additional file 1: Appendix A). After watching the clip, they were prompted to make the inference about what would happen next. After this they were given an 8 1/2” by 11” sheet of paper to draw a map from memory with the instructions printed at the top and grid lines for the map. They had 5 minutes to complete their map.

#### Data analysis

Predictive inference coding procedures were identical to experiment 1. Experiments 2a and 2b had high interrater reliability. For both Cohen’s *Kappa* = 0.954, *p* < 0.001. Discrepancies were resolved through discussion.

To score the maps, we used the automated Gardony Map Analyzer (Gardony et al., 2015). This starts with a master configuration map given as input to the software with all relevant locations labeled. Then, each participant’s drawn map is scanned, input to the software, its labeled locations are marked, and it is compared to the master map and given a similarity score. We used the SQRT (canonical accuracy) measure in the Gardony Map Analyzer (Gardony et al., 2015), which is a general measure that scores both on the number and configuration of landmarks. To create the master configuration map, Google Earth was used to find the actual streets (in Venice, CA, USA) on which the opening scene of *Touch of Evil* was filmed.^4^ Within the layout of the actual street, each of the locations in the clip was placed as accurately as possible to its location on the Google Map. This gave us an objectively accurate map of the scene, which would give participants who drew the most accurate maps the highest scores using the map analyzer.

### Results

#### Overview

As with experiment 1, the results of experiment 2a showed a strong comprehension effect. Bayes dfactors again showed that the majority of eye-movement measures supported the null (saccade lengths, gaze similarity, region of interest). However, the Context participants had longer fixation durations, and there was a targeted effect of looks at the car with the bomb the first time it is on the screen for No-context participants. During this time Context participants are more likely to look at the car than No-context. Overall, these results generally support the Tyranny of Film, but indicate there may be localized effects of comprehension on eye movements.

Experiment 2b introduced the map task to test if the Tyranny of Film could be turned off. Fixation duration, gaze similarity, and region of interest results all showed differences between the comprehension and map task conditions, which showed a break in the Tyranny of Film. There was not a complete dissociation in the attentional selection between the two conditions; thus, these results indicate that a task at odds with comprehension (i.e., the map task) can turn down the Tyranny of Film. These results show our measures are sensitive to differences in eye movements, and as a result that our null effects based on comprehension differences are likely true null effects (i.e., not the result of a weak manipulation or insensitive measures).

### Experiment 2a Results

#### Predictive inference

The results of this analysis largely replicated the results of experiment 1 with the Context condition more likely to make a bomb-relevant inference (*X* ^2^ (1, *N* = 193) = 46.39, *p* < 0.001, η = 0.490). Almost exactly half of the participants in the Context condition made a bomb-relevant inference (65 participants made the inference and 66 did not). The decrease in the frequency of inference from experiment 1 could be attributed to the absence of audio. This afforded a cleaner analysis comparing the eye movements of participants in the context conditions that did or did not generate the prediction. No participants in the No-context condition made the inference. Thus, data in all the following analyses are in terms of three groups: the Context participants that made the inference (Context + Inference), the Context participants that *did not* make the inference (Context + No-inference), and the No-context group.

#### Eye movements

##### Fixation durations and saccade lengths

Data cleaning followed the same procedure as experiment 1. The effects were reversed when compared to experiment 1. There was a marginal effect of group on fixation duration (*F* (2, 191) = 3.79, *p* = 0.024, ηp2 = 0.038; intersaccadic cleaning, *F* (2, 191) = 2.869, *p* = 0.059). The Bayes factor shows anecdotal evidence for the null (BF_01_ = 1.55). To describe the relationship, Tukey HSD post hoc comparisons for the analysis without intersaccadic interval cleaning indicated that the Context + Inference group had the shortest average fixation duration, which was significantly shorter (*p* = 0.026) than the No-context group (Table 1). The Context + No-inference group was not different from either of the other groups. There were no differences in average saccade length (*F* (2, 191) = 0.905, *p* = 0.406, ηp2 = 0.009; intersaccadic cleaning, *F* (2, 191) = 1.602, *p* = 0.204), with substantial evidence of the null shown by the Bayes factor (BF_01_ = 8.56). Descriptively, the Context + Inference group had longer saccade lengths than both the Context + No-inference group and the No-context group (Table 1). Interestingly, the magnitudes of the mean saccade lengths for the Context + Inference and No-context groups were very similar to those in experiment 1, which showed a significant difference, but a small-to-medium effect size. The only differences in these results between experiments 1 and 2 were that the mean saccade length of the No-context group increased from 4.63° (experiment 1) to 4.69° (experiment 2a), and the SDs for both groups were larger in experiment 2. In sum, the small effect of context on saccade amplitudes was replicated from experiment 1, but our caution in interpreting the effect in experiment 1 appears to have been justified as it failed to reach statistical significance this time.

Shorter fixation durations for the Context group that made the inference match the effect between fixation durations and comprehension during reading. Specifically, fixation durations tend to be shorter when a person has a better understanding for what they are reading (Rayner, 1998). In the current experiment, participants in the Context condition that made the inference show a high level of comprehension for the narrative and potentially shorter fixation durations. An alternative explanation is that Context participants may be exploring the scene more, which is usually associated with shorter fixation durations (Pannasch et al., 2008; Smith & Mital, 2013). The comparison of the effects of context and inference on mean fixation durations and mean saccade lengths in experiments 1 and 2 shows a pattern of small effects which slightly change between experiments, but are roughly the same. Because these effects are small, they can vary from being statistically significant to not significant, or vice versa, from one experiment to the other. Overall, we must treat these results with caution.

##### Gaze similarity

**Fig. 4:**
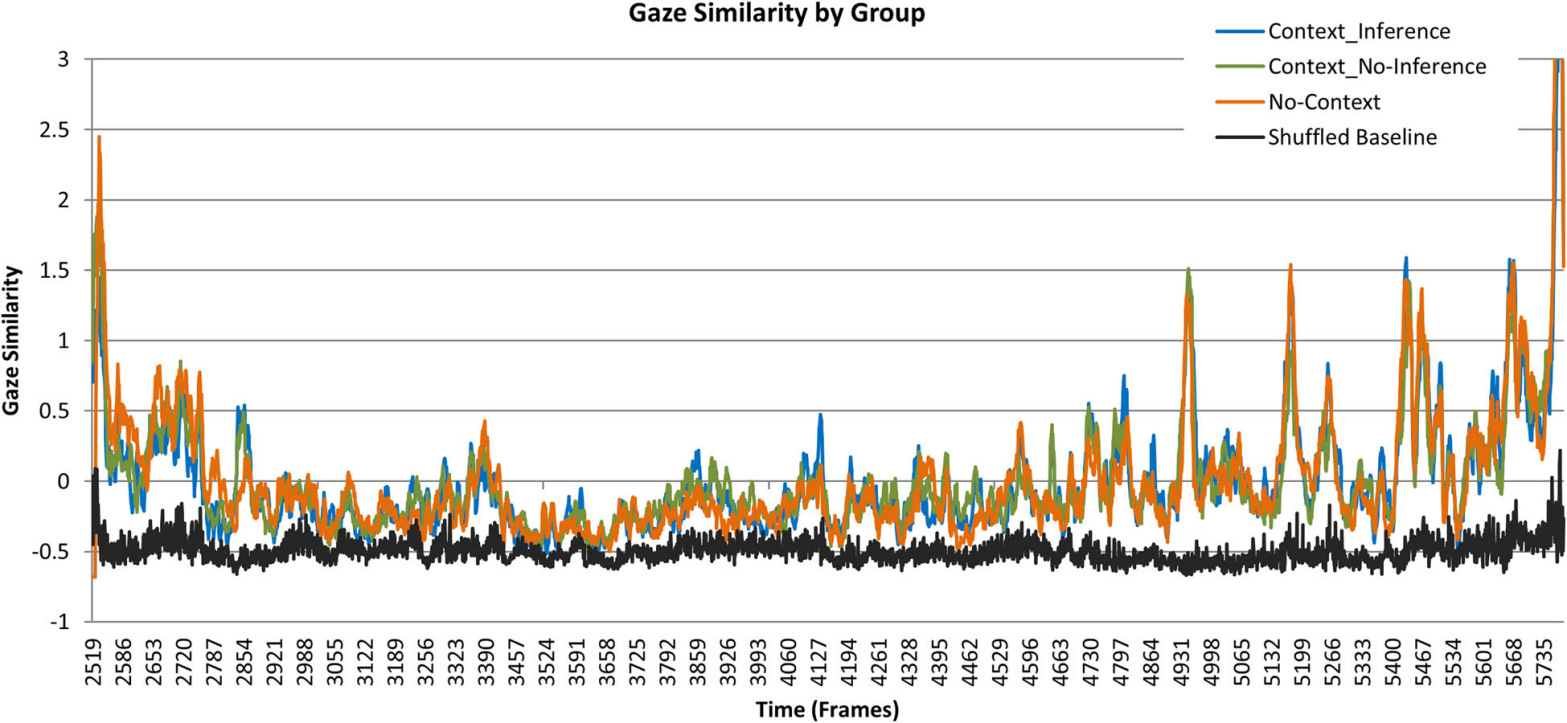
Similarity of gaze by context condition across the shared viewing period of the clip that starts on frame 2519 (thus, the figure appears different from Fig. 2). Gaze similarity is expressed as a z-score probability relative to the context condition and inference made (Context + Inference [*blue*], Context + No-Inference [*green*], No-context [*orange*], and shuffled baseline [*black*]). Larger values indicate greater attentional synchrony. The shuffled baseline (*black*) indicates chance level gaze similarity for the clip

The same data pre-processing procedure from experiment 1 was used for experiments 2a and 2b. As with experiment 1, the first analysis run was an omnibus test for gaze differences between the Context and No-context groups across the viewing of the critical portion of the film clip (the same 1 minute and 49 seconds of the clip that both groups saw). During this critical portion of the clip, there were no group differences (*F* (2, 190) = 0.05, *p* = 0.955). The Bayes factor showed strong evidence for the null (BF_01_ = 18.07), replicating experiment 1. Figure 4 shows that the lines indicating gaze similarity for each group are nearly identical, indicating that the tendency for viewers to look at the same places at the same times was the same across groups. Again, a shuffled baseline was included for comparison for the shared viewing period. Overall, the gaze similarity of all three experimental groups was generally above the shuffled baseline, indicating that the film was guiding eye movements, potentially creating the Tyranny of Film. Including the shuffled baseline in the ANOVA was statistically significant (*F* (3, 254) = 55.23, *p* < 0.001, ηp2 = 0.395), with all experimental groups higher than the random baseline, also replicating the results of experiment 1. In addition, Fig. 4 shows that experiment 2 replicated the systematic peaks and troughs of gaze similarity across the entire film clip, including the familiar peak of gaze similarity at the end of the clip (previously shown in Fig. 2c) when the walking couple kiss. Thus, despite replicating a null effect of context on gaze similarity, the gaze similarity measure was highly sensitive to changes in attention across the film clip.

These results indicate that viewers’ understanding of the clip did not influence their gaze similarity. Even when viewers knew about the bomb or thought the car and the couple in it were the main characters of the film clip, they viewed the clip similarly to viewers who did not. However, we cannot explain this null effect in terms of the film clip failing to guide eye movements because overall gaze similarity was significantly and meaningfully well above chance for all three groups, and systematically related to the film content (e.g., the kiss). Thus, the film did influence viewers’ attention very systematically, but the differences in viewers’ comprehension between groups had no influence on their gaze similarity.

##### Region of interest

The same region of interest data pre-processing as in experiment 1 was used. The car was identified as the region of interest, and we tested whether our three viewing groups differentially looked at it. The region of interest analyses again started using the entire shared viewing period, and then an a priori time point of interest based on the manipulation of protagonists/agents.

**Fig. 5:**
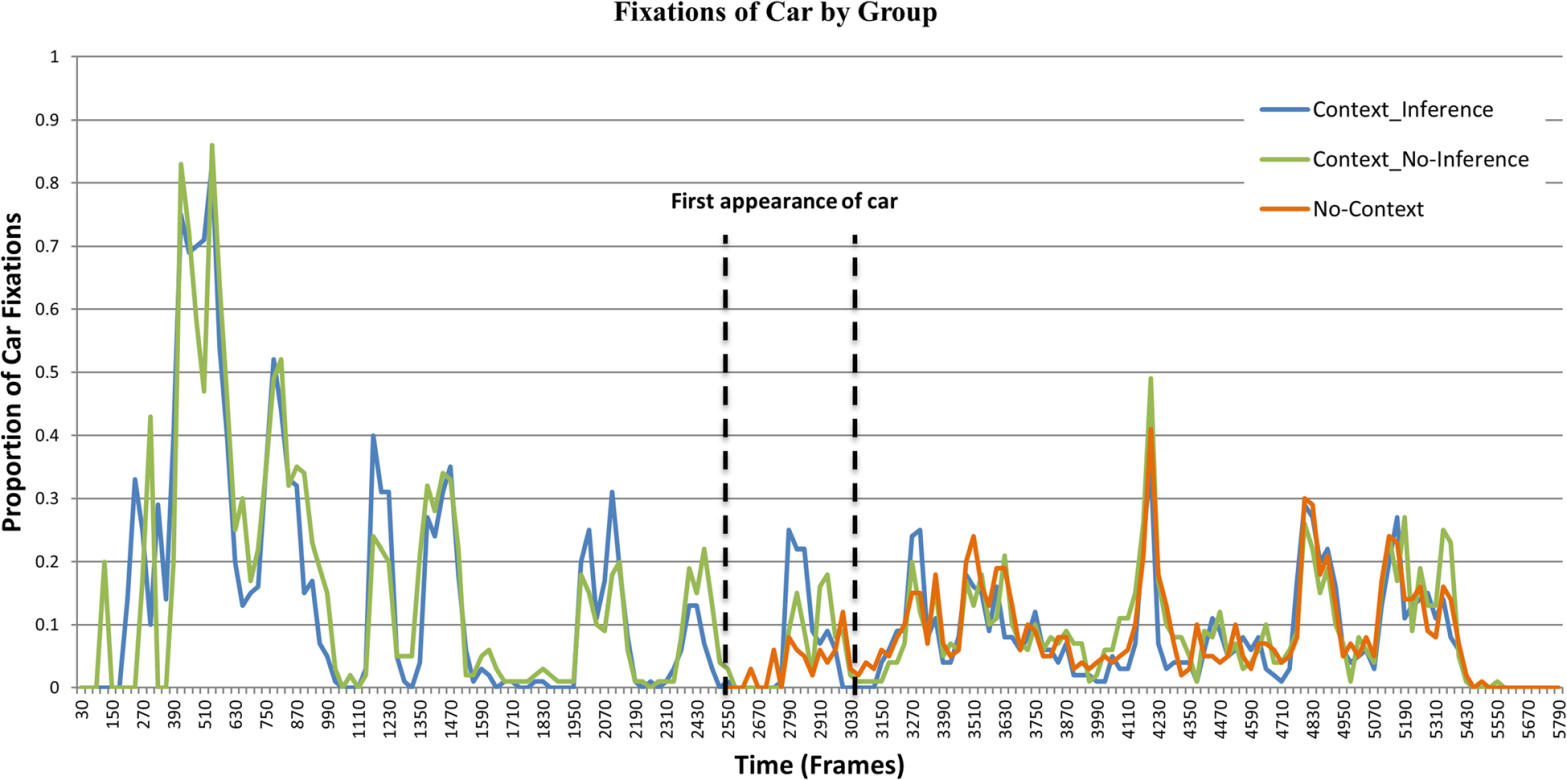
Proportion of participants fixating the car by context condition and inference throughout the film clip (Context + Inference [*blue*], Context + No-inference [*green*], and No-context + No-inference [*orange*]). The higher the value, the more participants looking at the car. The first appearance of car for No-context group is marked with *vertical dotted lines*

The omnibus region of interest analysis started the first time the car appeared on the screen in the No-context condition, 1 minute and 57 seconds into the film clip. Overall, there were no significant differences between the three groups in how often they fixated the car during this viewing period (*F* (2, 190) = 1.07, *p* = 0.345; Fig. 5). There was strong evidence for the null hypothesis (BF_01_ = 7.40). This is in line with the gaze similarity analysis, indicating that the manipulation of both knowledge of the bomb and the protagonists had no effect on viewers’ overall likelihood of looking at the car.

We next carried out a more specific a priori region of interest analysis to probe a critical time period when the manipulation of protagonist might be expected to have an effect on fixations of the car. This was the 8-second period when the car with the bomb was first seen by participants in the No-context condition. At that point in the narrative, the walking couple walks past the car, which is stationary because a crowd of pedestrians has blocked the street. For viewers in the No-context condition, the car should have no particular importance, but for viewers in the Context condition, it should already be an integral component of their event model, regardless of whether the bomb is still active in their event model or not. Thus, in line with the eye–mind hypothesis (Just & Carpenter, 1980; Reichle, Pollatsek, Fisher, & Rayner, 1998; Reilly & Radach, 2006), the event model hypothesis would predict that the Context condition viewers would be more likely to look at the car than the No-context condition, at least initially. To test this prediction, we created an 8-second time window from when the car first appeared (frame 2758) and right before it was briefly occluded from view again (frame 3028), and we then measured the proportion of fixations of the car for each group. A one-way (group, Context + Inference vs. Context + No-inference vs. No-context) between subjects ANOVA found a main effect of viewing group on proportion of fixations on the car during the pre-specified 8-second time window (*F* (2, 190) = 3.93, *p* = 0.021, ηp2 = 0.04). As illustrated in Fig. 5, the Tukey HSD procedure indicated that viewers in the No-context group were significantly less likely to fixate the car (M = 0.054, SD = 0.078) than those in the Context + Inference group (M = 0.098, SD = 0.086) (*p* = 0.02). There was also a non-significant trend (*p* = 0.06) for viewers in the Context + No-inference group to fixate the car less than viewers in the Context + Inference group (M = 0.093, SD = 0.122). This provided the first support for the influence of the event model on predictable gaze behavior in the *Touch of Evil* film clip. Viewers who knew about the bomb and already had the car in their event model were more likely to fixate the car during its first appearance within the critical period than participants who had not indexed the car (and the couple in it) as protagonists, or more generally, important agents in the narrative. We will refer to this as evidence of the “agent effect”, as it seems to be due to whether viewers treat an entity in the narrative as an “agent” or not.

In general, experiment 2a showed support for the Tyranny of Film with there being no overall differences in gaze distribution across the different inference conditions. The fixation duration effect may indicate that Context + Inference participants are under less cognitive load than No-context participants, which is consistent with what is expected for participants with better comprehension (Rayner, 1998), but we treat this result with caution. The agency effect, reported for the region of interest analysis demonstrates the potential for gaze to be influenced by an object’s relevance to the viewer’s event model, but the fleeting nature of this effect suggests that the motivation for such top-down control may need to be stronger and more deliberate during film viewing than has been demonstrated in static scene viewing (DeAngelus & Pelz, 2009; Smith & Mital, 2013; Yarbus, 1967) due to the bottom-up saliency of film*.* Given our pattern of null effects of comprehension (i.e., context and inference) on eye movements, experiment 2b was conducted to test whether it was possible to get strong positive effects of cognition on eye movements with our stimuli and measures. This tests whether the previous experiments are showing a true overall null effect of comprehension on eye movements, or if there may simply be a problem with our stimuli or measures.

### Experiment 2b Results

Eye-movement analyses compared participants in the map task to participants in the Comprehension group. The Comprehension group contained only those participants in the Context condition who generated the predictive inference, because those participants demonstrated the filmmaker’s intended comprehension of the scene. The Map task group participants were those at or above the median map score who did not make the predictive inference about the bomb. These two groups represent participants that most successfully completed their task: to comprehend the narrative or to create a mental map of the scene.

#### Predictive inference

Inference data were initially analyzed for all 75 participants that completed the map task. The inference results for the Map task condition were compared to those of the Comprehension group. Participants in the Map task condition were less likely to make the inference about the bomb (mean proportion = 0.13) than those in the Comprehension group (mean proportion = 0.50, (*X* ^2^ (1, *N* = 205) = 27.56, *p* < 0.001, η = 0.367). Rerunning the analysis for only the Map task participants that were included in the eye-movement analyses based on their map score showed the same effect (mean proportion = 0.10, (*X* ^2^ (1, *N* = 171) = 20.974, *p* < 0.001, η = 0.350). This indicates that the map task was cognitively at odds with the process of narrative comprehension (i.e., participants had identical visual information available, including seeing the bomb put in the car, but processed it differently to complete their given tasks). The key question is whether part of the difference in processing was the deployment of overt visual attention as measured by eye movements.

#### Eye movements

##### Fixation durations and saccade lengths

All data were cleaned using the same procedures as outlined in experiment 1. For fixation durations, there were no significant differences between the groups. In the Comprehension group the average fixation duration was descriptively longer (Table 1) than the Map task group, but not statistically significant (*t* (100) = 0.695, *p* = 0.489; intersaccadic cleaning, *t* (100) = 0.672, *p* = 0.503). There was strong evidence of no difference in mean fixation durations between the Comprehension and Map task groups (BF_01_ = 5.27).

Mean saccade length between groups, however, did show a significant difference. Consistent with our hypotheses based on the results of Lahnakoski et al. (2014) and Smith and Mital (2013), average saccades were significantly longer in the Map task group than in the Comprehension group (Table 1) (*t* (100) = 4.56, *p* < 0.001; *d* = 0.91; intersaccadic cleaning, *t* (100) = 3.349, *p* < 0.001; *d* = 0.66). We hypothesized that this would occur because the Map task participants would make longer saccades in order to explore the edges of the scene to complete their task, thereby (at least partially) ignoring the main characters of the narrative that are typically shown near the center of the screen, which would require shorter saccades to explore.

##### Gaze similarity

**Fig. 6:**
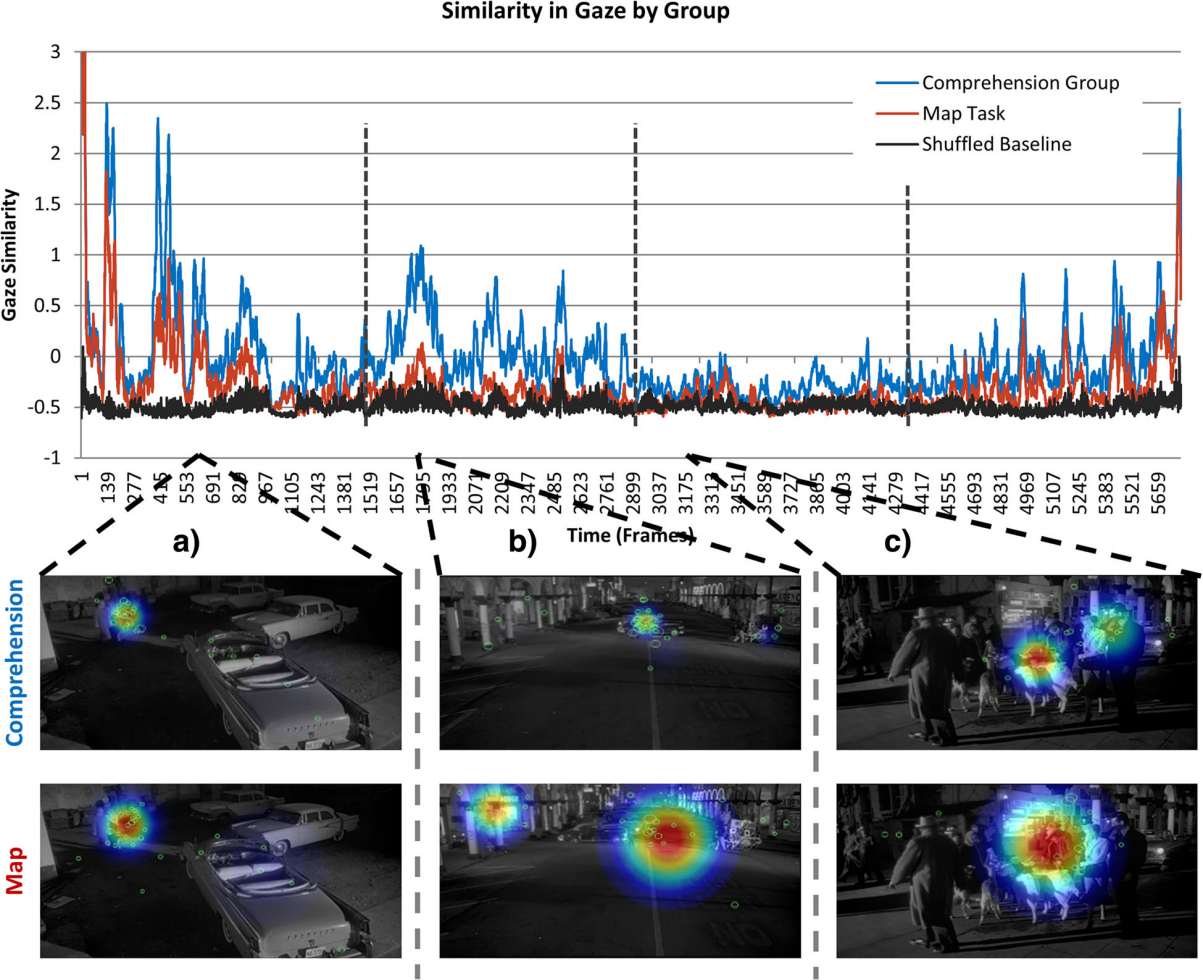
*Top*: Similarity of gaze by context condition across the full clip. Gaze similarity is expressed as a z-score probability relative to the context condition and inference made (Comprehension group [*blue*], Map task [*red*], and shuffled baseline [*black*]). Large values indicate greater attentional synchrony. *Vertical dashed grey lines* illustrate the quarters used in the repeated measures analysis. *Bottom*: The stills exemplify gaze patterns during high gaze similarity (GS) for both groups (**a**), high GS for Comprehension, low for Map Task (**b**), and low GS for both (**c**)

The gaze similarity analysis compared Comprehension group participants to those in the Map task group across the entirety of the film clip. Figure 6 shows the results of this comparison.

Qualitatively, Fig. 6 shows that gaze similarity scores for the Comprehension group are often higher than for the Map task group, indicating that viewers in the Map task group were looking at different places on those given frames than the Comprehension group. To quantify this relationship an ANOVA of mean gaze similarity by group was calculated. Consistent with the qualitative assessment of the figure, an ANOVA supports the difference between each group (*F* (2, 166) = 96.484, *p* < 0.001, ηp2 = 0.541), with Bonferroni-corrected pairwise comparisons showing the Comprehension group had the greatest gaze similarity (M = 0.001, SD = 0.266), followed by the Map task (M = −0.284, SD = 0.234), and the shuffled baseline (M = −0.488, SD = 0.045) was the lowest. Nevertheless, Fig. 6 also shows that the Map task group was frequently above the shuffled baseline, and mimicked many of the peaks and troughs of the Comprehension group. This supports the prediction that even when the task is at odds with narrative comprehension, it may be difficult to completely ignore areas associated with the narrative. The Tyranny of Film is perhaps not being turned off, but instead is being turned down.

In addition to the main effects, an inspection of Fig. 6 indicates that there may be an interaction of gaze similarity with time. An exploratory, repeated measures ANOVA of task and time in the clip was conducted to better understand the role the features of the clip play in determining gaze similarity. As noted in the “Background”, we chose the *Touch of Evil* film clip, in part, because it has bottom-up filmic features that should reduce attentional synchrony. However, the presence of visual features that may guide attention changes throughout the clip. For the repeated measures ANOVA, the clip was broken into quarters that correspond well to the changes in visual features in the clip (more detail on these features in the interpretation below). The sphericity assumption was violated (*X* ^*2*^ (5) = 36.077, *p* < 0.001); thus, a Greenhouse-Geisser correction was used (*Ɛ* = 0.869). As with the one-way ANOVA, there was a main effect of time block (*F* (2.60, 427.76) = 177.113, *p* < 0.001, ηp2 = 0.519). Bonferroni-corrected pairwise comparisons showed that each time block was significantly different from the others; block 1 had the highest gaze similarity (M = −0.114, SD = 0.419), followed by block 2 (M = −0.227, SD = 0.355), then block 4 (M = −0.266, SD = 0.313), and block 3 had the lowest gaze similarity (M = −0.406, SD = 0.151). Additionally, the task by time block interaction was significant (*F* (5.217, 427.758) = 71.430, *p* < 0.001, ηp2 = 0.466). The interaction was probed using simple effects where time block was held constant over task. The Greenhouse-Geisser corrected omnibus error term was *MSE* = 0.016, and the *df* = 427.758. Gaze similarity differed between all tasks in blocks 1, 2, and 4 (*p* values < 0.001, *F* values > 20.312; see Additional file 1: Appendix C for full simple effects structure). However, in block 3, the Map task and the shuffled baseline were not significantly different (*F* (1, 427.758) = 2.375, *p* > 0.05), while all other task comparisons in block 3 were significantly different (*p* values < 0.001, *F* values > 34.187).

The main effect of time block and the interaction with task support previous work on the features that guide attentional synchrony when a qualitative analysis of the features of a film clip in each block is used. As shown in Fig. 6 (top), block 1 has the highest gaze similarity, and qualitatively has features that would support this (Fig. 6, bottom, a; e.g., close ups of the bomb and car, and relatively little else to look at). Figure 6 shows that blocks 2 and 4, which have moderate levels of gaze similarity, also have moderately more to look at (Fig. 6, bottom, b; e.g., more store fronts). Block 3 has the lowest gaze similarity, and the most complex composition involving lots of people, vehicles, goats, and store fronts (Fig. 6, bottom, c). The complexity in block 3 is precisely what we predicted would reduce attentional synchrony overall when we chose the *Touch of Evil* film clip (Bazin, 1967; Cutting et al., 2011; D’Angelo, 2012; Mital et al., 2010; Yarbus, 1967). Additionally, the large number of storefronts and spread out locations in block 3 is what would be predicted to reduce gaze similarity in the map task, consistent with the lack of a difference between the Map task group and the shuffled baseline in block 3.

##### Region of interest

The same region of interest data pre-processing as in experiment 1 was used. The car was identified as the region of interest, and we tested whether participants in the Comprehension group fixated the car more often than in the Map task group. The region of interest analysis was carried out over the entire viewing period.

**Fig. 7:**
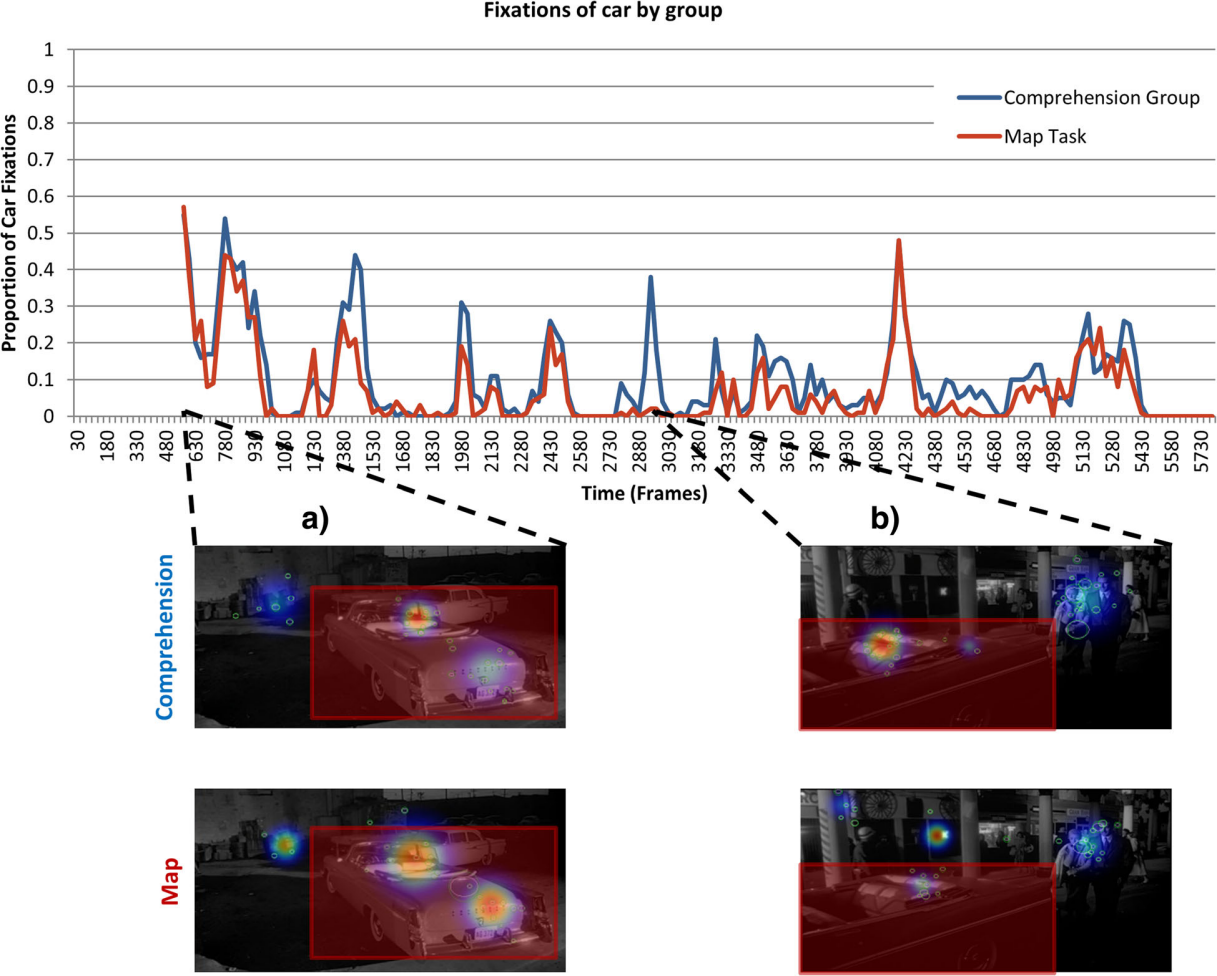
*Top*: Proportion of participants fixating the car throughout the film clip (Comprehension group [*blue*], Map task group [*red*]). The higher the value, the more participants looked at the car. The car first appears in the clip at frame 541, which is in time bin 570. *Bottom*: Film stills show the region of interest for the frame, with fixation and heat maps superimposed. The stills indicate when both groups fixated the car at a similar rate (**a**) and when the car first reappears and was highly fixated by the Comprehension group, but not the Map task group, who looked at the storefronts (**b**)

As can be seen in Fig. 7, the red line for participants that completed the map task is fairly consistently below that of the blue line for the Comprehension group. A *t*-test comparing the proportion of fixations of the car (when on the screen) for each group confirmed that participants in the Comprehension group fixated the car significantly more often (10.2% of fixations) than participants in the Map task group (6.5% of fixations) (*t* (100) = 3.706, *p* < 0.001; *d* = 0.74). A similar result was shown when overall dwell time on the car was calculated for each group. As with the proportion of fixations, the total time spent on the car was larger for participants in the Comprehension group (16.9 s, SD = 10.8 s) than for the Map task group (11.6 s, SD = 7.1 s) (*t* (100) = 2.653, *p* = 0.009; *d* = 0.53). Thus, the comparison of the map and comprehension tasks created a medium-to-large effect for looks at the car.

Taken together, the region of interest results are consistent with our predictions for the map task. The car is integral for comprehension of the film clip, but is relatively unimportant for completing the map task. Accordingly, participants in the Comprehension group looked at the car more than participants in the map task. As a result, the region of interest results again show the Tyranny of Film being reduced through the high level cognitive task manipulation of the map task. Nevertheless, as with the gaze similarity analysis above, participants in the Map task condition still looked at the car occasionally, indicating that the reduction of the Tyranny of Film was relative, but not complete.

### Discussion

Experiment 2a tested the role of context on comprehension and eye movements, and experiment 2b tested the role of task. The results of 2a showed a limited effect of comprehension, as manipulated by context, on eye movements. That limited effect was primarily during a critical period when there was a difference in the perceived agents in the film, which affected visual attention. To test whether the Tyranny of Film could be *turned-off* by an explicit task at odds with comprehending the film narrative, experiment 2b compared the Context + inference group with the Map Task condition. The results of this comparison showed a statistically significant and meaningful reduction in both the gaze similarity and probability of looking at the chief region of interest (the car) due to the task manipulation. Thus, we can say that an explicit viewing task that was at odds with the task of comprehension *turned-down* the Tyranny of Film. Because we showed experimentally induced variation in the degree of the tyranny of film, this suggests that our previously shown dissociation between comprehension (as manipulated by context) and eye movements is a *true null effect* rather than being due to a weak manipulation of comprehension or poor measures of the cognitive effects on eye movements. This is something that will need further testing, as the likelihood that it is a true null effect can only be ascertained through a programmatic approach of attempts to reject the null, such as the current study. Importantly, these results also suggest that the online control of eye movements in dynamic scenes is highly task dependent.

## General discussion

Why does viewing and understanding films seem so easy? Some have argued that this happens because filmmakers have mastered the craft and over the past century have developed a set of practices that direct attention (Smith, 2012) and comprehension (Magliano, Dijkstra, & Zwaan, 1996; Magliano, Miller, & Zwaan, 2001; Magliano & Zacks, 2011). Filmmakers have documented this notion of control over how viewers process film (Lumet, 1995; Murch, 2001). However, until recently few studies have explored how these techniques guide the attentional processes that affect the earliest stages of comprehension (Loschky et al., 2015). The results of the present study are largely consistent with those of Loschky et al. (2015) and support the Tyranny of Film hypothesis. The intuitions that cinema conventions guide the eye, and in turn comprehension, are born out in the present study. Moreover, we extend insights gained from Loschky et al. (2015), who focused on a clip from a James Bond movie, *Moonraker*, that used intensive continuity editing practices (Bordwell, 2002). It makes sense that there is strong attentional synchrony in such a film because the length of the shots and the dynamic movement do not afford top-down driven exploration (Smith, 2012). Interestingly, a long continuous shot, such as that used in the present study, potentially does allow endogenous exploration, especially in the context of a situationally rich environment, as in the scene from *Touch of Evil*. Nonetheless, our results showed a remaining Tyranny of Film that was likely produced by the filmmaker’s mis-en-scene (i.e., staging), framing of the shot, and movement of the camera in the scene, drawing viewers’ eyes where the filmmaker likely intended.

### Rethinking the tyranny of film

**Fig. 8:**
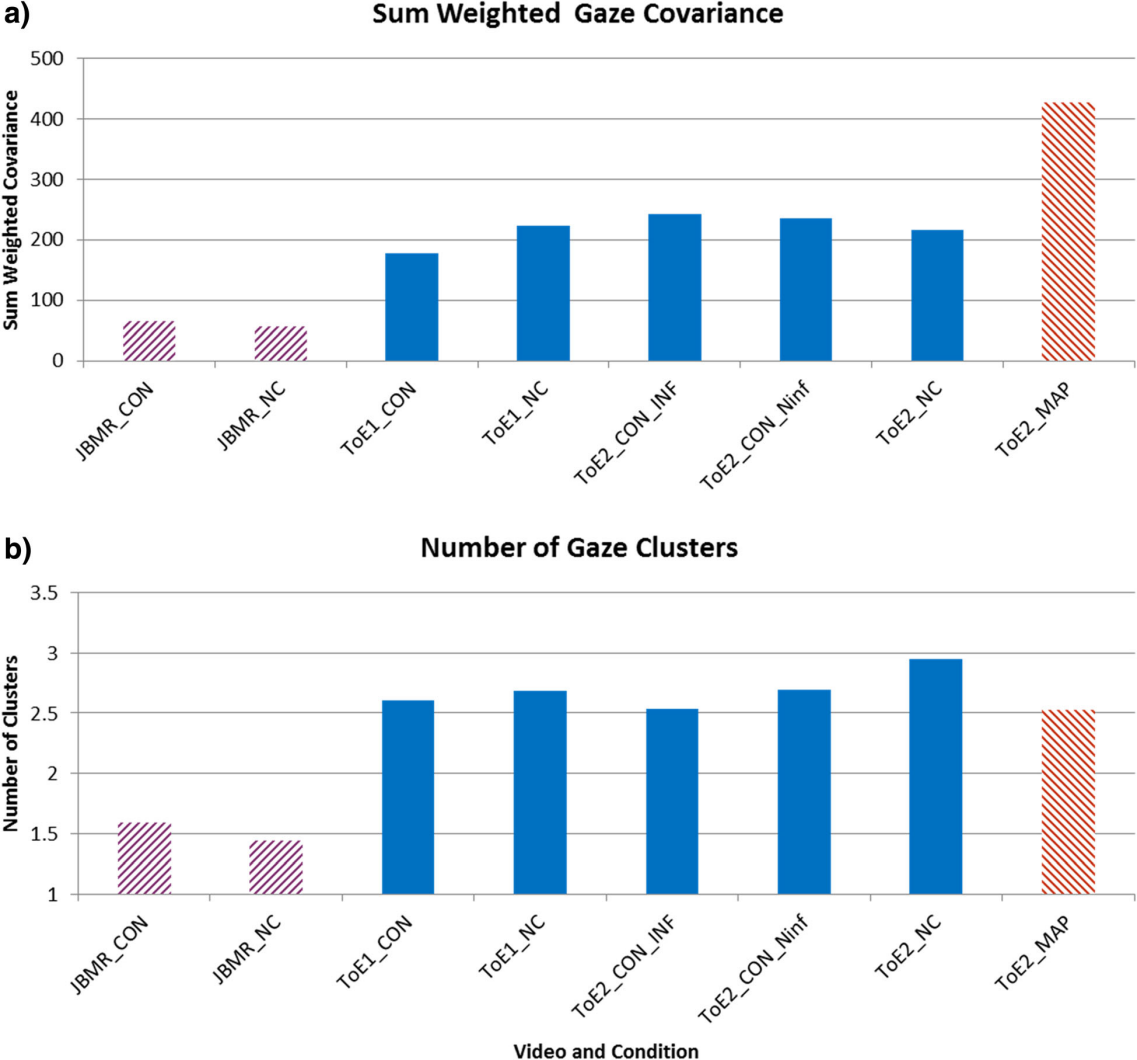
**a** The average sum weighted gaze covariance for all eye-tracking conditions in the James Bond film *Moonraker* (Loschky et al., 2015) and *Touch of Evil* studies. The *purple bars* are for the *Moonraker* study, the *blue bars* are for the *Touch of Evil* comprehension conditions, and the *red bar* is for the *Touch of Evil* map task. **b** The average number of gaze clusters for the same experiments. *JBMR_CON*, James Bond *Moonraker* Context condition; *JBMR_NC*, James Bond *Moonraker* No-context condition; *ToE1_CON*, *Touch of Evil* experiment 1 Context condition; *ToE1_NC*, *Touch of Evil* experiment 1 No-context condition; *ToE2_CON_INF*, *Touch of Evil* experiment 2 Context + Inference; *ToE2_CON_Ninf*, *Touch of Evil* experiment 2 Context + No-inference; *ToE2_NC*, *Touch of Evil* experiment 2 No-context; *ToE2_MAP*, *Touch of Evil* map task

One potential reason for the overall null effect of comprehension on eye movements with *Touch of Evil* (Welles & Zugsmith, 1958) could be that even though it was chosen for what appeared to be weak bottom-up features, it still guided participant eye movements as much as the *Moonraker* (Broccoli & Gilbert, 1979) film clip used in the previous study (Loschky et al., 2015). However, Fig. 8a, b clearly show *Moonraker* produced more clustering (i.e., lower sum of weight gaze covariance) on fewer clusters than *Touch of Evil*. The covariance measure presented below is the sum of the covariances of the optimal number of clusters used to describe the distribution of the gaze during each frame. The “weighted” component of the measures indicates that the covariance measure gives more weight to clusters that are composed of more gaze points (see Mital et al., 2010 for more details). This is essentially the amount of spread in gaze for each frame, while controlling for the number of clusters of gaze and how many participants are in each cluster. Furthermore, as would be expected based on the gaze similarity results, the map task had even less clustering than all other conditions.^5^ Thus, it seems that even films with relatively weak bottom-up features show little effect of comprehension on eye movements. *Touch of Evil* does have relatively weak bottom-up features when compared to *Moonraker*, yet the comprehension and eye-movement results are analogous.

The results in Fig. 8 call for a reconsideration of the Tyranny of Film hypothesis, which states that there is no opportunity for differences in comprehension to be expressed through differences in eye movements due to bottom-up guidance of viewer attention by visual features. Clearly, fewer people are looking at the same places at the same times in *Touch of Evil* than in *Moonraker*, yet we still find few effects of the large differences in comprehension (as manipulated by context) on eye movements.

The results in Fig. 8 also allow us to more fully consider the gaze similarity differences between the Comprehension and Map task groups shown in Fig. 6. Those results showed that, as predicted, the Map task group had reduced gaze similarity compared to the Comprehension group. However, the gaze similarity metric by itself cannot tell us if that reduced gaze similarity was due to an overall reduction in attentional synchrony for the Map task group, or to equal attentional synchrony but at a different screen location(s) than the Comprehension group. In fact, as noted above, Fig. 8 indicates that the Map task group indeed had reduced attentional synchrony (i.e., more sum-weighted gaze covariance, and fewer gaze clusters). Finally, Fig. 7 showed that the Map task group specifically looked less at the car than the Comprehension group, which may account, at least in part, for the reductions in attentional synchrony (Fig. 8), and gaze similarity (Fig. 6).

### Top-down attention takes time in film

One possible reason for the modest relationship between film comprehension and eye movements during film viewing is that there is often a delay in top-down effects when the scene is not known before onset (Carmi & Itti, 2006; Smith & Mital, 2013). The *Touch of Evil* clip was chosen in part because it doesn’t introduce a new shot every 2–3 seconds, but it may be that the introduction of new information due to the camera continuously tracking in the shot creates a similar effect of keeping the viewer in an early stage of processing that relies more on bottom-up processing. While it is now well known that, when viewing static scene images, there are immediate top-down effects on eye movements (e.g., the first saccade in a visual search task; Eckstein et al., 2006; Ehinger, Hidalgo-Sotelo, Torralba, & Oliva, 2009; Torralba et al., 2006), the same has not been found when viewing dynamic scenes, namely film and video. Instead, when viewing video with no prior knowledge of its content, top-down effects on attention have been found to have a delayed onset of 2–3 seconds, which has largely been explained as being due to the overwhelming attentional capture by motion during that early time period (Carmi & Itti, 2006; Smith & Mital, 2013). Further work is needed to test the differences between task-specific and comprehension-based differences in top-down effects on visual attention when viewing film and video.

The notion that top down effects may take time is consistent with research on establishing coherence in discourse comprehension at the local level (i.e., how the current narrative constituent fits in with the immediate context) versus the global level (i.e., how the current narrative constituent fits in with the larger story context). There was considerable debate as to whether readers need to establish global coherence (a top-down process) in light of evidence of local coherence (Graesser, Singer, & Trabasso, 1994; McKoon & Ratcliff, 1992). However, that debate has largely been resolved and there is strong evidence that readers routinely establish both local and global coherence (e.g., O’brien & Albrecht, 1992). In the context of reading narratives, global coherence can be supported by memory-based activation (McKoon & Ratcliff, 1998; Myers & O’Brien, 1998). Upon reading a sentence, semantically aligned content from the mental model is passively activated, which can then be used to establish global coherence. Importantly, the output of those processes can take time and be completed while reading subsequent sentences (O’Brien & Cook, 2016). However, in the case of the present study, if one does not have propositions reflecting knowledge of the bomb being associated with the car stored in the mental model for the scene, then obviously that knowledge cannot serve as a basis for establishing global coherence when the car re-appears in the street.

### When comprehension has an effect

Overall, our study found few effects of comprehension on eye movements during film viewing. However, we found the agent effect in experiment 2a, and modest effects of the event model were also found in Loschky et al. (2015). Thus, film comprehension at the local level (i.e., current event model construction) can have an impact on visual attention during film viewing. Interestingly, the effects found may have occurred through different mechanisms. The agent effect found in the current study appears to have occurred because participants in the No-context condition continued to track the agents (the walking couple) in their event model, which may have been a more automated process (Findlay & Walker, 1999). Conversely, the effect in Loschky et al. (2015) appears to have occurred due to the No-context group needing to use effortful processing to update their event model with what was presented. Based on this, differences in eye movements may be indicative of the amount of effortful processing the viewer used to comprehend a scene. The map task similarly required effortful searching and mental map updating to successfully complete the task. The importance of the potential need for effortful processing is that it may indicate the relative difficulty of updating the various event indices of an event model during narrative comprehension (Zwaan, Langston, & Graesser, 1995). In the current study, we only manipulated two of the five indices of the event indexing model (agent and causality). A more comprehensive study manipulating all five indices (agent, time, place, causality, and intentionality [goal-relevance]) may give a clearer picture of the effect of each of these event indices on attention. We hypothesize that when an event index must be effortfully updated to maintain comprehension for a narrative, it will predictably guide eye movements.

### What breaks the Tyranny of Film?

Despite the ambiguity of what drives the Tyranny of Film, the relative dissociation between eye movements and narrative comprehension is very surprising in *Touch of Evil*, especially given the strength and consistency of the differences in comprehension between the groups. These findings are inconsistent with the majority of previous work looking at top-down task-based effects on scene viewing (Foulsham & Underwood, 2007; Henderson et al., 2007; Henderson et al., 2013; Smith & Mital, 2013; Yarbus, 1967).

One characteristic of this finding is that it is inconsistent with a strict interpretation of the eye–mind hypothesis (Just & Carpenter, 1980; Reichle et al., 1998; Reilly & Radach, 2006). It appears that the event index of causality, in relation to the bomb, in participants’ event models is not guiding eye movements, while agency is having a modest effect. One way of explaining these results is in terms of the above-mentioned local and global levels of coherence. Perhaps the process of identifying and locating key agents in the narrative, and attending to them, is an important local coherence maintenance process, as suggested by the agent effect found in experiment 2a. Agent tracking may operate at a lower level than the maintenance and calculation of causal relationships (e.g., potential consequences of the bomb), which may be considered more of a global coherence maintenance process. According to this idea, during film viewing, local processing of the narrative may strongly guide eye movements, while global processing of the narrative may have a weaker effect. This would not necessarily suggest that global processing of causal event indices cannot affect eye movements. It is possible that, for example, because the bomb was hidden in the trunk of the car, a look at its hiding place provided no new information.^6^ Nevertheless, this interpretation of the results would argue for a weaker version of the eye–mind hypothesis. Namely, depending on viewers’ level of coherence processing (i.e., local vs. global) there may be stronger or weaker associations between eye movements and thought (Lamont, Henderson, & Smith, 2010; Smith, 2015).

This weak version of the eye–mind hypothesis may be due in part to film viewing being driven by both bottom-up features (Mital et al., 2010) and *mandatory* top-down processes (Baluch & Itti, 2011). Mandatory top-down processes are well-learned, more automated processes, as opposed to volitional top-down processes. A classic mandatory process is the hollow face illusion, when a concave face (e.g., the inside of a mask), is perceived as convex (Baluch & Itti, 2011; Gregory, 1970). Another example is following the speaker of a conversation (Birmingham et al., 2008; Coutrot & Guyader, 2014; Ho et al., 2015; Vo et al., 2012). Within film viewing, the task of comprehension may have certain mandatory processes used to construct an event model, such as agent tracking. According to this idea, causality maintenance and calculation may be less automatic than agent tracking, and may require more volitional top-down attentional control.

One recent theory that is consonant with the above hypotheses is the role of the default mode network in narrative comprehension (Tylén et al., 2015). That is, the default mode network may allow for the accumulation of coherent plot information. Conversely, when plot information is less coherent, the frontoparietal control network is thought to allow for a more effortful search for narrative coherence through a more top-down deployment of attention. The reason for this is that the default mode network has been shown to be less active when visual attention is effortfully deployed (Andrews-Hanna, Reidler, Huang, & Buckner, 2010; Andrews-Hanna, Reidler, Sepulcre, Poulin, & Buckner, 2010). One might expect that the default mode network should not play a large role in processing the highly complex visual stimulus of a film, but fMRI research on film viewing has found that it activates the default mode network (Hasson et al., 2008; Hasson, Malach, & Heeger, 2010). Additionally, areas thought to make up part of the default mode network have been shown to be similarly activated during both film viewing and audio book listening (Hasson et al., 2008; Yeshurun et al., 2017).

Based on the above, while watching a movie, breaking the Tyranny of Film may have two potential paths. The first could be to directly tap into a mandatory process (e.g., agent tracking) that may be necessary for maintaining local coherence. If the narrative one viewer perceives in a scene has entirely different characters than the narrative another perceives in the same scene, they should track different agents. We tested this hypothesis in the current study, but the film clip used appears to have been well constructed by the filmmakers to give high importance to both the walking couple and the couple in the car; thus, the observed agent effect was short-lived. The other track to breaking the Tyranny of Film is to move away from mandatory processing and automated comprehension processes. The map task appears to have done this, but it should be possible with a comprehension manipulation as well. For example, in Loschky et al. (2015), the effect on eye movements occurred during a complex cross-cutting sequence that required viewers to make an inference (that both sequences would come together in time and space and solve the life-and-death problem faced by the protagonist in one of the two sequences). The viewers that had more trouble making the inference about a critical shot showed eye-movement differences during that shot. However, this shot was essentially a static scene, and thus lacked important motion features to guide viewer attention. Thus, future work should test if a break in coherence allows viewers to move from mandatory processing to more effortful, volitional processing even during dynamic scenes in a film.

### Implications for theory and practice

The opening scene of *Touch of Evil* was chosen in part because it is one of the mostly widely discussed examples of a long-take in film history. It is theorized to illustrate how the craft of filmmaking can guide attention and affective response (D’Angelo, 2012), but to also allow for differences between the viewers (Bazin, 1967, p. 35–36). As such, it was an ideal clip for testing the role that the viewer’s active film comprehension processes play in driving their attention.

The results of this study speak to the long-standing debate in film theory regarding the nature of meaning and how it is derived. Classic perspectives on film assume that the structure of film plays a central role in how films are understood (Eisenstein, 1948; Pudovkin, 1970). The Spielberg (2013) quote at the outset of this paper illustrates this perspective. On the other hand, constructivist perspectives of film response assume that meaning is (almost) entirely derived by the viewer, and there is no inherent connection to content (Hall, 1980), a view voiced by Tarantino in the second quote at the beginning of this paper. Similar debates exist in a variety of areas of cognitive science, such as regarding top-down and bottom-up contributions to perception (Firestone & Scholl, 2016) and to comprehension (Graesser et al., 1994).

Overall, the present results are consistent with a growing view in film theory that the structure of a film matters for meaning making (Anderson, 1998). We have learned from experiments 1 and 2a that despite large differences in global understanding of a film, the visual features of film will guide the attention of different viewers to the same things at the same times. We assume that what viewers attend to will have an impact on what knowledge is activated moment-to-moment as a film is processed. Experiment 2b provided evidence consistent with this by showing that participants in the comprehension task overtly attended to more narrative-relevant content (e.g., the car) than those in the map task, and subsequently showed better comprehension of the narrative (as indicated by a much higher probability of making a bomb-relevant inference). Knowledge activation provides the basis for mental model construction (Kintsch, 1988, 1998; Myers & O’Brien, 1998). If viewers have the goal to understand and be entertained by a film, their mental models will likely be closely constrained by that activated knowledge (Graesser et al., 1994). Such results are consistent with the central role of the film in guiding viewers’ attention and understanding of it (Bordwell & Thompson, 2001; Katz, 1991; Murch, 2001). However, if viewers have an idiosyncratic goal when watching a film, the constructive processes that support that activity could lead to a mental model that reflects ideas beyond the events conveyed in the film. The results for the map task are consistent with this assertion, since viewers who did well in the task came away after watching the clip with a non-normative representation of it (centered on its spatial layout, rather than its suspense-inducing narrative). Such results are consistent with the constructivist perspective (Bordwell & Carroll, 1996; Hall, 1980; Tseng & Bateman, 2013).

### Some considerations regarding the nature of the present study

The scene from *Touch of Evil* was chosen for this study for very specific reasons. It is one of the most well discussed and described scenes of its era precisely because it is seen as a textbook example of how cinematic techniques can affect how films are processed (Bazin, 1967). Specifically, it is considered a virtuoso example of creating and maintaining a high level of suspense throughout a three-minute opening of a film (D’Angelo, 2012). This allowed us to manipulate the presence or absence of that suspense (i.e., a vivid example of film comprehension). In addition, the clip is equally famous for having a single three-minute shot with no cuts, extremely rare in film history (Bazin, 1967), thus allowing us to test our previous proposal (Loschky et al., 2015) that the weak effects of comprehension on eye movements while watching a film clip was due to using as series of short (~ 2 s) shots.

However, we must acknowledge the fact that the conclusions based on this study are grounded in using one film clip. While this limits the generalizability of our results to some extent, the results are both consistent with and build upon those of Loschky et al. (2015), which used a very different film clip. Furthermore, there are numerous widely cited studies that pioneered entirely new areas of research that used a single complex naturalistic stimulus (Hasson, Nir, Levy, Fuhrmann, & Malach, 2004; Yarbus, 1967), a single real-world interaction (Simons & Levin, 1998), or, when studying high level comprehension, of a single narrative (Anderson & Pichert, 1978; Bransford & Johnson, 1972). There are good reasons for this. When using such complex naturalistic stimuli as *found materials*, they often differ on a variety of dimensions beyond those that are the target of any given study, and those differences can introduce confounds that can make using multiple, naturally occurring items untenable given the goals of the study. Our current study, and those cited above, are prime examples of this point. Of course, a program of research cannot rest on a series of studies based on single items. Nevertheless, we see such studies as playing a valuable role in the seminal stages of a program of research because they can guide the generation of critical hypotheses to be tested with more controlled and generalizable studies. As such, as the study of film processing matures, it is likely that more naturalistic materials (e.g., clips from existing films) will have to give way to more controlled, experimenter-generated materials with numerous versions sharing features of interest (for a similar argument, see Magliano & Graesser, 1991).

## Conclusions

The current study tested whether a person’s comprehension during film viewing affects their attention, as measured by their eye movements. The differences in comprehension we found were similar to those commonly found in studies of reading comprehension, but rarely studied with film. However, despite these large comprehension differences, similar to our previous study that used a very different film clip (Loschky et al., 2015), we found only modest and targeted differences in eye movements. These findings are counterintuitive based on work looking at top-down effects on eye movements in static scenes (Yarbus, 1967), but consistent with the finding of strong attentional synchrony in film viewing (Dorr et al., 2010; Mital et al., 2010; Smith & Mital, 2013; Wang et al., 2012). Based on these results, the Tyranny of Film hypothesis was mostly supported. This suggests that the processes by which information is extracted through eye movements may differ between film narratives and reading (Magliano et al., 2013), as seen in the general dissociation between eye movements and comprehension in our study. The results are interesting in terms of both film comprehension processes and eye-movement processes in film perception, but the dissociation of these processes may be of most interest. During film viewing, people can look at the same places at the same times but have different understandings of the narrative. These results seem to support both the views expressed by Spielberg and Tarantino in the quotes at the beginning of this paper. This is a counter-example to the common assumption in many eye-movement studies that there is a strong association between eye movements and thought (Just & Carpenter, 1980; Reichle et al., 1998; Reilly & Radach, 2006). Nevertheless, we did find an effect on eye movements of a manipulation of local coherence (the agent effect) that was stronger than our manipulation of global coherence (the causal consequences of the bomb). Further studies should investigate whether such local versus global coherence manipulations differentially affect eye movements during film viewing. Furthermore, the results from the Map task condition in experiment 2b indicate that tasks at odds with film narrative comprehension can provide evidence for cognitive control of eye movements, supporting the eye–mind hypothesis. To better understand these relationships between eye movements and comprehension during film viewing, further studies need to combine theories and methods from the fields of scene perception, event perception, and narrative comprehension (Loschky, Hutson, Magliano, Larson, & Smith, 2016; Loschky, Hutson, Magliano, Larson, & Smith, 2014; Magliano, Larson, Higgs, & Loschky, 2016).

Importantly, this research drew on the craft knowledge of the filmmakers of the clip used and theory about the clip and film more generally. As such, the current study can inform the theory and practice of filmmaking. We started the paper with two contrasting quotes from prominent filmmakers. Our results provided more evidence consistent with Spielberg’s perspective, in that our participants mostly knew “…where to look at the exact same time” (Spielberg, 2013). However, as we have discussed, demonstrating control over gaze during film viewing does not guarantee control over comprehension and affective response. In fact, our research shows that there can be a disconnect between them. In addition, we show evidence of film viewers’ ability to intentionally take an idiosyncratic approach to attending to and understanding the film clip, showing that different viewers can, with effort, see a movie very differently than intended, consistent with Tarantino’s perspective. Thus, our research shows how both Spielberg and Tarantino can be correct, under different viewing conditions, even though on the surface their quotes appear contradictory. Further research will be needed to test and refine these hypotheses.

## Additional file

Additional file 1: Appendix. (PDF 480 kb)

## Abbreviations

BF_01_: Bayes factor
CARPE: Computational and Algorithmic Representation and Processing of Eye-movements
*d*: Cohen’s *d*
JBMR_CON: James Bond Moonraker Context condition
JBMR_NC: James Bond Moonraker No-context condition
M: Mean
NSS: Normalized scanpath saliency
SD: Standard deviation
ToE1_CON: *Touch of Evil* experiment 1 Context condition
ToE1_NC: *Touch of Evil* experiment 1 No-context condition
ToE2_CON_INF: *Touch of Evil* experiment 2 Context + Inference
ToE2_CON_Ninf: *Touch of Evil* experiment 2 Context + No-inference
ToE2_MAP: *Touch of Evil* map task
ToE2_NC: *Touch of Evil* experiment 2 No-context

## Footnotes

1 These errors values are based on the calibration procedure, and the technical specifications for the EyeLink1000 from SR Research. Our video clips were shorter in duration than the tests done by SR Research to produce those specifications, and would not be expected to require recalibration.

2 Additional support for comprehension differences were found through event segmentation (Loschky et al., 2016; Loschky et al., 2014; Magliano et al., 2016) results (Zacks & Tversky, 2001). The event segmentation results indicate online differences in segmentation, with viewers in the No-context condition identifying more event boundaries and boundaries at different locations than those in the Context condition.

3 This dialogue was, at most, a minor problem for experiment 1. First, the mention is not specifically of the bomb, but rather of a “ticking noise”. This presumably is what allowed exactly one participant out of 42 (i.e., 2%) in the No-context condition in experiment 1 to make an inference about a bomb, but the vast majority, 41 of 42 (98%) in experiment 1 did not. The one thing it most likely did was remind Context condition participants about the bomb, which would explain the higher inference rate for that condition in experiment 1 over 2. Second, the mention of the bomb occurs with only 15 s left in the clip, so even if it did remind Context condition participants of the bomb, this would not have an effect for the first 2 minutes and 39 seconds of shared viewing time in experiment 1.

4 Numerous *Touch of Evil* fan websites discuss the filming location of this classic film, and particularly the famous opening scene filmed in Venice Beach, California. Based on one such site (Loschky et al., 2014), and our own judgment comparing the film with current and archival photos of the area, we chose the streets on Google Maps to use. The shot starts at a lot near the corner of Windward Ave. and Speedway. From there the shot goes south west on Woodward Ave. to what is now Ocean Front Walk. From there the shot goes north west on Ocean Front Walk.

5 The lack of a difference in the number of clusters for the map task and the other *Touch of Evil* conditions makes sense, because as the overall clustering lessens, there are going to be fewer groupings of gaze that are identified as a cluster.

6 Of course, since about half of the viewers who knew about the bomb did not make a predictive inference about it at the end of the clip, this suggests that they were unable to hold it in their working memory till the end of the clip (and data to test this hypothesis support it, as shown in Hutson et al. (Robh, 2011)). Yet, those participants (Context + No-inference) were just as likely to look at the car as those who did not forget the bomb (Context + Inference), or those who never knew about the bomb (No-context + No-inference).
