# Supplementary material for: What is the role of the film viewer? The effects of narrative comprehension and viewing task on gaze control in film
Source: Cogn Res Princ Implic. 2017 Nov 22;2:46. doi: 10.1186/s41235-017-0080-5 (PMC5698392; doi:10.1186/s41235-017-0080-5)
Supplement: Supplementary file 1 — Appendix. (PDF 480 kb) [file 41235_2017_80_MOESM1_ESM.pdf]

Appendix

A)

Map Task Instructions:

Your task is to ***watch a video*** *clip of a town, and after you finish,* ***draw a map*** *of the area depicted from memory. Your map should be as detailed as possible* ***including naming and labeling*** *as many locations as possible. Your map will be scored for its level of detail and accuracy. You have 5 minutes to complete your map.*

B)

Example Maps (Experiment 2b)

Low Map Score (0.0)

a) Ambiguous Labels

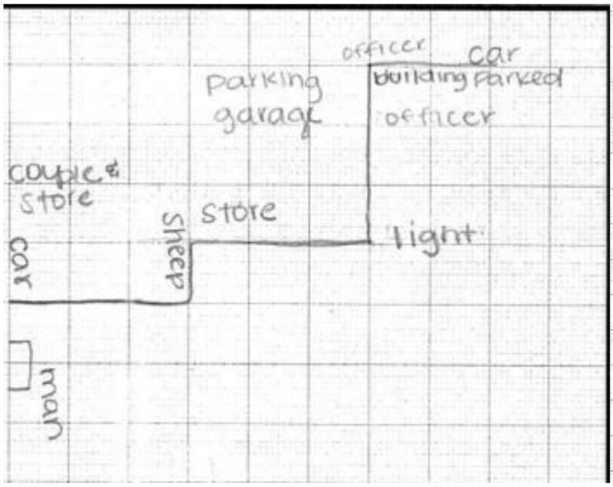

b) Locations not identified

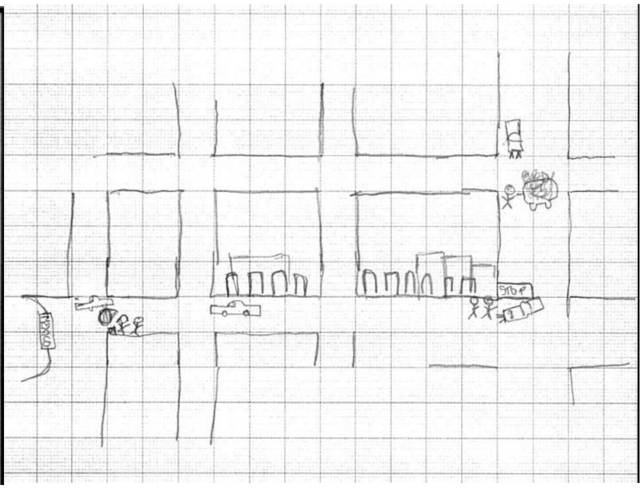

Median Map Score (.17)

a) 4 locations labeled

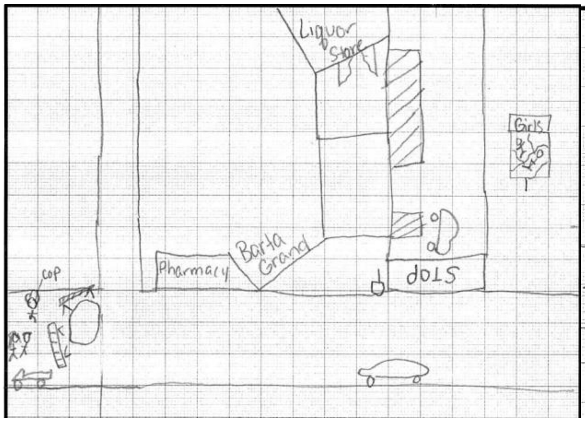

b) 4 locations labeled

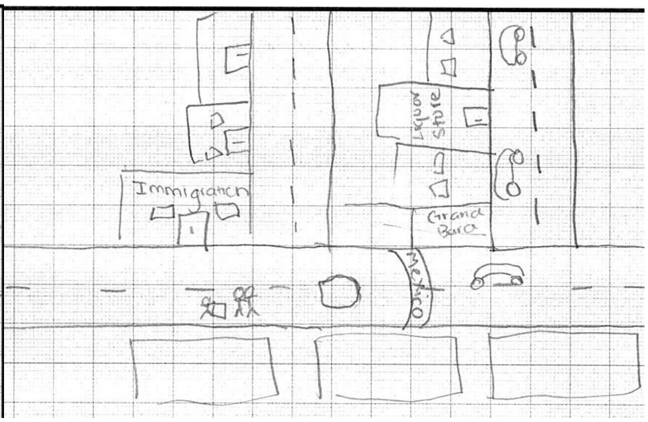

Top-Scores (.37 & .38)

a) .37 (7 locations given near correct location)

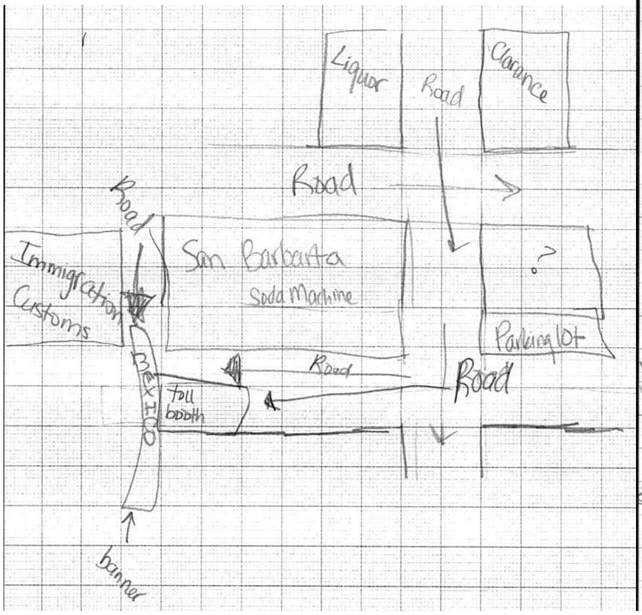

b) .38 (7 locations given near correct location)

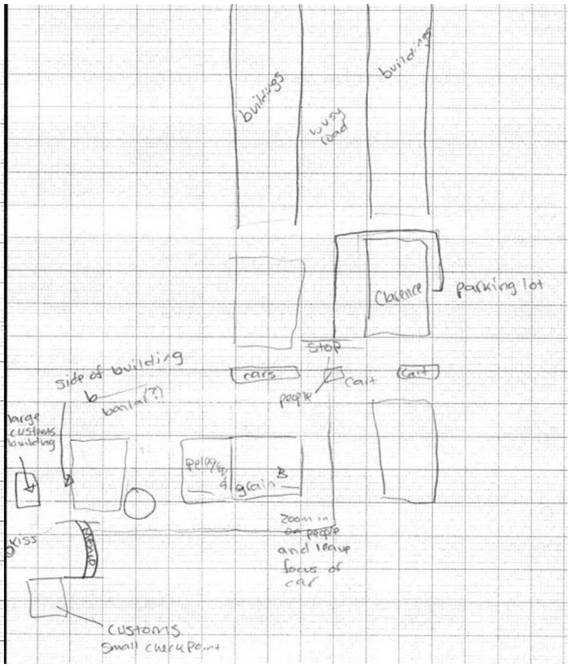

C)

Experiment 2b: Gaze Similarity Repeated Measures ANOVA

Simple Effects:

Quarter held constant over condition:

The omnibus error term was .016 and the degrees of freedom were 427.758.

Quarter 1:

- Context and Map: F = 2.417/.016; F (1, 427.758) = 151.062, p <.001
- Context and Shuffle F = 18.020/.016; F (1, 427.758) = 1126.25, p <.001
- Map and Shuffle F = 4.248/.016; F (1, 427.758) = 265.5, p <.001

Quarter 2:

- Context and Map: F = 4.426/.016; F (1, 427.758) = 276.625, p <.001
- Context and Shuffle F = 9.857/.016; F (1, 427.758) = 616.062, p <.001
- Map and Shuffle F = .325/.016; F (1, 427.758) = 20.312, p <.001

Quarter 3:

- Context and Map: F = .547/.016; F (1, 427.758) = 34.187, p <.001
- Context and Shuffle F = 1.204/.016; F (1, 427.758) = 75.25, p <.001
- **Map and Shuffle F = .038/.016; F (1, 427.758) = 2.375, p > .05**

Quarter 4:

- Context and Map: F = 1.254/.016; F (1, 427.758) = 78.375, p <.001
- Context and Shuffle F = 7.059/.016; F (1, 427.758) = 441.188, p <.001
- Map and Shuffle F = 1.307/.016; F (1, 427.758) = 81.688, p <.001

D)

Heat Map Videos

https://www.youtube.com/playlist?list=PLChGnR0Bh6QUhGEN7TPbfNK08zGYkMCn3
